# Decoding the southeastern Tibetan Plateau growth: a 3D numerical simulation of Cenozoic crustal deformation

**DOI:** 10.1093/nsr/nwag118

**Published:** 2026-02-27

**Authors:** Yuyang Wang, Yang Wang, Jianfeng Yang, Lijun Liu, Jinjiang Zhang, Peizhen Zhang

**Affiliations:** Guangdong Provincial Key Laboratory of Geodynamics and Geohazards, School of Earth Sciences and Engineering, Sun Yat-Sen University, Guangzhou 510275, China; Guangdong Provincial Key Laboratory of Geodynamics and Geohazards, School of Earth Sciences and Engineering, Sun Yat-Sen University, Guangzhou 510275, China; State Key Laboratory of Lithospheric Evolution, Institute of Geology and Geophysics, Chinese Academy of Sciences, Beijing 100029, China; State Key Laboratory of Lithospheric Evolution, Institute of Geology and Geophysics, Chinese Academy of Sciences, Beijing 100029, China; The Key Laboratory of Orogenic Belts and Crustal Evolution, School of Earth and Space Sciences, Peking University, Beijing 100871, China; Guangdong Provincial Key Laboratory of Geodynamics and Geohazards, School of Earth Sciences and Engineering, Sun Yat-Sen University, Guangzhou 510275, China

**Keywords:** southeastern Tibetan Plateau, numerical modeling, Cenozoic tectonic evolution, kinematic transition, geodynamics

## Abstract

The southeastern Tibetan Plateau, an intracontinental deformation archetype recording oblique Indian–Eurasian convergence, has long been used to test geodynamic models of plateau growth. Driven by India’s northward indentation, it has undergone multiphase deformation with kinematic and structural transitions. To explore its evolutionary dynamics, we developed 3D visco-elasto-plastic thermomechanical models reconstructing three tectonic stages: (i) crustal shortening; (ii) block lateral extrusion; and (iii) kinematic reversal in the southeastern Tibetan Plateau. Simulations show that strain localization along large-scale shear zones is initially controlled by lithospheric heterogeneities enabling rigid block extrusion. Since the mid–late Miocene, vertically stratified crustal rheology has promoted decoupling, in which potential energy-driven ductile lower crustal flow affects upper crustal deformation and triggers kinematic reversal. This transition reconciles block extrusion and lower crustal flow, which operate sequentially rather than exclusively and are modulated by temporal variations in crustal rheology and boundary conditions, resolving the long-lasting debate of geodynamics during continental collision.

## INTRODUCTION

**Figure 1.**
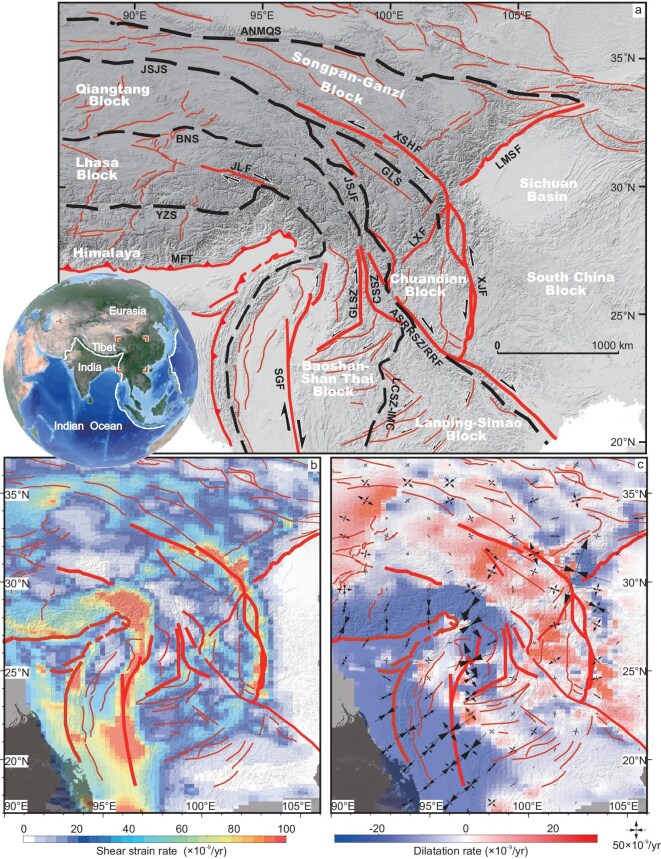
Tectonic framework and modern crustal deformation of the southeastern Tibetan Plateau. (a) Main fault systems and structures in the study area, with red lines representing major active faults and black dashed lines representing suture zones. MFT, Main Front thrust; SGF, Sagaing Fault; ASRRSZ, Ailaoshan–Red River shear zone; RRF, Red River Fault; XJF, Xiaojiang Fault; LMSF, Longmenshan Fault; XSHF, Xianshuihe Fault; JLF, Jiali Fault; YZS, Yarlung Zangbo suture zone; BNS, Bangong–Nujiang suture zone; JSJS, Jinshajiang suture zone; ANMQS, Anemaqen suture zone; GLS, Ganzi–Litang suture zone; GLSZ, Gaoligong shear zone; CSSZ, Chongshan shear zone; LCSZ-IMC, Lincang shear zone–Inthanon metamorphic complex. (b) Present-day shear strain rate derived from the GNSS velocity field [47]. (c) Present-day dilatation strain rate derived from the GNSS velocity field [47], with black cross lines indicating the principal strain directions.

The uplift and expansion of the Tibetan Plateau record the intracontinental dynamics driven by the India–Eurasia continental collision, providing crucial clues for understanding the mechanisms of crustal deformation and mountain building [1,2]. The southeastern Tibetan Plateau is sandwiched between the northward advancing Eastern Himalayan Syntaxis (EHS) and the stable South China Block (Fig. 1a). This region, which experienced multistage Tethyan accretionary orogenesis, plays a crucial role in adjusting the lateral expansion of the Tibetan Plateau during the Cenozoic [3,4]. The ongoing oblique continental convergence has led to intensive crustal thickening, complex fault systems, and topographic uplift. In response to the early collision, a giant compressional–transpressional zone developed from the Malay Peninsula northward to Yunnan Province, China. Shortening-related structures such as folds, thrusts and inversion structures in Meso–Cenozoic sedimentary basins are widespread; compression, anatectic metamorphism and multistage magmatic emplacement are well documented within the exhumed mid–lower crust [5–8]. Since the Oligocene, several continental-scale shear zones, including the Ailaoshan–Red River, Chongshan–Lincang–Inthanon and Gaoligong–Mogok shear zones, have initiated to accommodate block extrusion and rotation, delineating the first-order tectonic framework in this region, and leading to the widely cited ‘escape tectonics’ [3,4,9]. Striking changes in kinematics and deformation styles may have occurred in the mid–late Miocene. Slip sense reversed and right‐lateral movement commenced along the Red River Fault, as well as several parallel NW-striking faults. A set of NE-trending faults also experienced diachronous kinematic reversal from dextral to sinistral motion [4]. In addition, the regional deformation pattern also transformed from localized strain to later diffuse deformation [4,10].

Over the past half-century, a large amount of geological and geomorphologic observations, geochemical and chronological dating, geophysical analyses and geodynamic simulations have been conducted in this region, which have enabled us to gain a certain understanding of regional tectonic evolution, lithospheric deformation, landscape evolution and deep dynamic processes [3,11–18] (Fig. 1b and c). However, it still remains unclear when and how these large-scale strike–slip faults initiated and what roles they play in accommodating lithospheric and crustal deformation. Furthermore, the driving mechanisms for the regional tectonic transition and intracontinental orogeny are the subject of significant controversies. Two contrasting geodynamic models, including the rigid block extrusion model [11,19] and the lower crustal flow model, lie at the core of this debate. The rigid block extrusion model attributes crustal deformation to strain localization along boundary faults surrounding largely undeformed crustal blocks [11,19,20]. In contrast, the lower crustal flow model invokes gravitationally driven mid–lower crustal ductile flow to explain surface uplift, crustal thickening and distributed deformation. Structural and geological observations emphasize the dominant role of these continental-scale faults; however, current Global Navigation Satellite System (GNSS) and geophysical observations often favor distributed deformation and the existence of low-viscosity crust at depth [12,15–17,21]. Reconciling these end-member mechanisms remains elusive. A key limitation of previous studies is their predominant focus on the surface geometry, kinematics and chronology of Cenozoic deformation, with limited attention paid to quantitative geodynamic processes. Geodynamic numerical models that couple lithospheric strength, rheological heterogeneity and boundary conditions offer a powerful tool to resolve these issues [1,2,20,22–29].

In this study, we utilize 3D visco-elasto-plastic thermomechanical numerical models to investigate the Cenozoic tectonic evolution and crustal deformation of the SE Tibetan Plateau in response to the India–Eurasia convergence. Our model reconstructs a three-stage tectonic evolution including crustal shortening, block extrusion and subsequent kinematic reversal. We demonstrate that large-scale shear zones in the SE Tibetan Plateau were initially governed by lithospheric heterogeneities, and that block extrusion may have transitioned to crustal rotation around the EHS, likely accompanied by lower crustal flow since the mid–late Miocene. Our model results provide a unified framework to reconcile both localized strain and diffused deformation, aligning well with geological and geophysical observations, and offer new insights into the dynamic evolution of the Tibetan Plateau.

## RESULTS

### Numerical quantification of tectonic units

**Figure 2.**
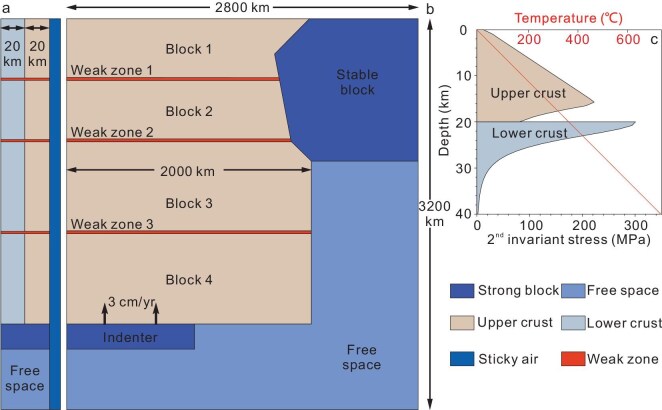
Model setup and crustal strength. (a and b) Left side view and top view of the model. (c) Crustal strength of the models in this study with the background strain rate of 10^−16^·s^−^^1^. The strength is defined by the depth integral of stress profile. In the upper crust, the strength mainly depends on plastic deformation. The yield stress associated with plastic deformation increases with depth. The temperature was set as a linear relationship, with the bottom surface temperature at 700°C. Both the stable block and the indenter used the material parameters of the strong block (see detailed material parameters in the Supplementary data).

The 3D thermomechanical numerical code LaMEM [30] is employed to quantitatively reconstruct the Cenozoic tectonic evolution of the SE Tibetan Plateau, which is based on a finite-difference staggered grid discretization combined with particle-in-cell techniques utilizing conservative velocity interpolation [23]. This code primarily solves the conservation equations for mass, energy and momentum via a geometric multigrid method (see details in the Methods). We use non-linear visco-elasto-plastic rheologies to simulate crustal deformation. Our study area is simplified into a region of 2800 × 3200 × 40 km (Figs 1 and 2). The crustal thickness is set at 40 km, consisting of a 20-km-thick upper crust and a 20-km-thick lower crust [31], with material parameters following previous studies [22] (Table S1; more model results are shown in the Supplementary Data). In the early Cenozoic, the NE Indo-Australian subduction zone might have been characterized by rapid slab rollback and large-scale trench retreat [16], which could have provided space for the extrusion of the SE Tibetan Plateau and the Indochina Peninsula. Therefore, the SE model boundary is set as a free boundary by using a weak material following the typical model of Tapponnier *et al.* [11,19]. To mimic an internal free surface in the finite-difference model, we introduce a 10-km-thick ‘sticky air’ layer [32], with a free-surface stabilization algorithm [33]. All other mechanical boundaries are free slip. The surface and basal temperature are set to 0°C and 700°C, respectively, with a linear temperature gradient throughout the model. We also set the basal temperature at Moho depth as 800°C and 900°C for comparison (see the details in Table S2). In the simplified model, we model the Indian Plate as a rigid indenter moving northward at 3 cm/year, consistent with GNSS-derived convergence rates relative to the northern boundary of the Songpan–Ganzi Block [34]. The South China Block is simplified as a relatively stable quasicraton polyhedron with rigidity the same as the rigid Indian Plate. Three rheologically weak zones represent the Bangong–Nujiang suture zone, Jinshajiang suture zone and pre-existing fault zones, respectively [35]. These weak zones thus divide the whole domain into four tectonic blocks: the Lhasa–Tengchong Block, Qiangtang–Indochina–Simao Block and South/North Songpan–Ganzi Block, respectively (Figs 1 and 2).

### Evolution of major shear zones and blocks

**Figure 3.**
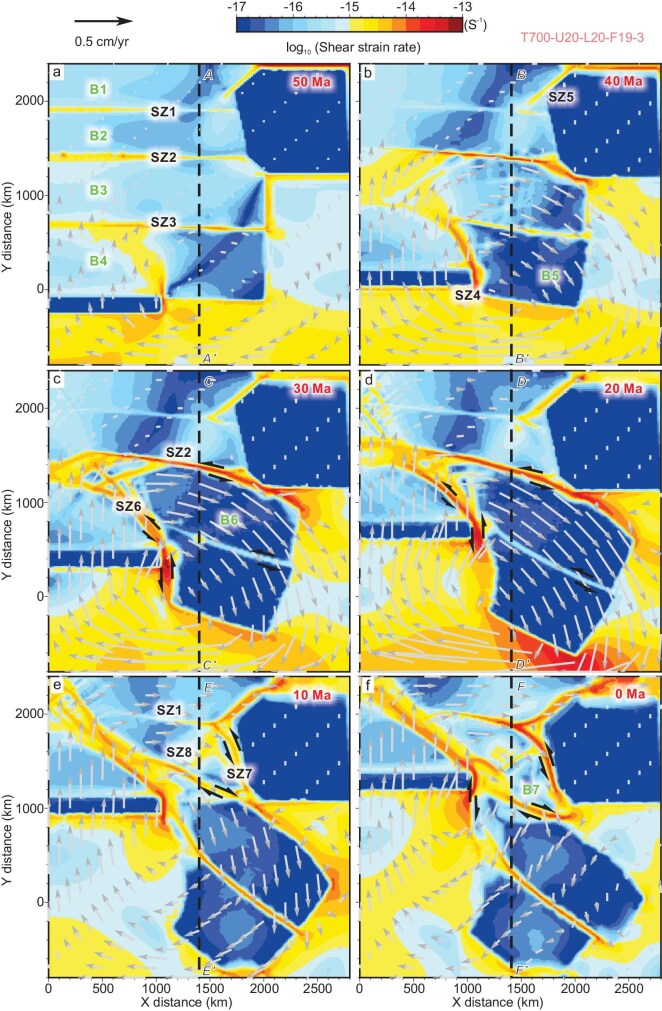
The evolution of strain rates at 2 km depth. The gray arrows represent the modeled velocity field. Red color denotes regions of high strain, while blue denotes regions of weak strain. The red time labels denote the corresponding geological time intervals of the model. (a and b) Initial collision induced higher strain along the weak zones (SZ1–3) with minimum deformation within blocks. (c and d) As the Indian Plate advanced northward, SZ4 and SZ6 formed, which are characterized by dextral movement. Large-scale sinistral displacement occurred along SZ2. They accommodated the southeastward extrusion of B5 and B6. SZ3 also shows sinistral displacement, adjusting the differential movement between B5 and B6. (e and f) Since the mid-late Miocene, SZ7 developed, merging with reactivated SZ1. SZ2 shifted to dextral motion, and rhomboidal B7 formed with clockwise rotation. The black dashed lines indicate the locations of the cross-sections in Fig. 5.

**Figure 4.**
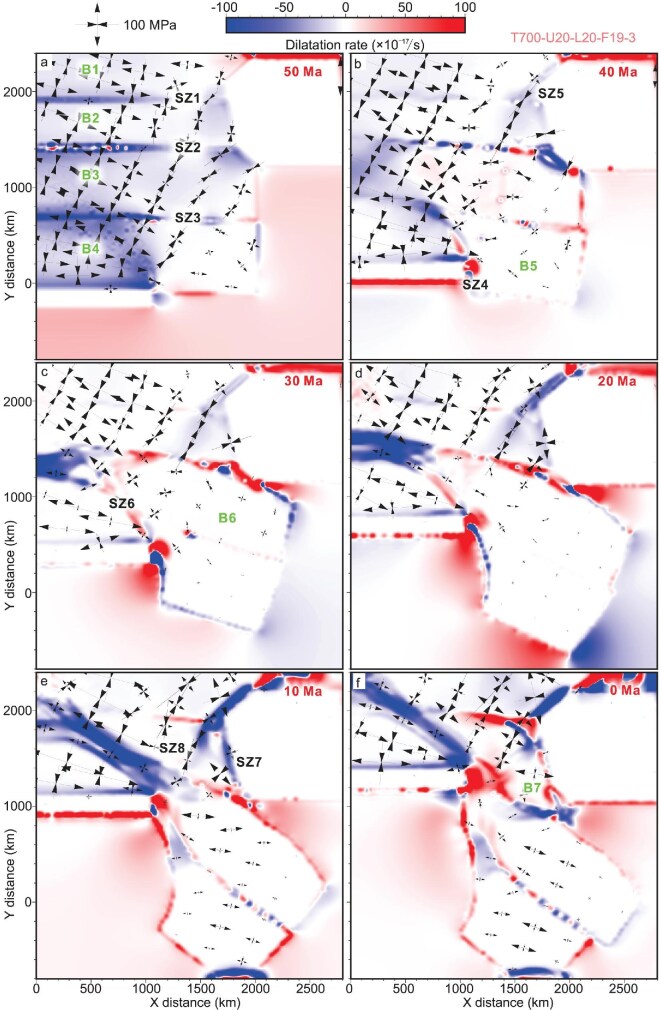
The evolution of dilatation strain rate at 2 km depth. Positive values of the dilatation strain rate indicate extensional deformation, while negative values indicate compressional deformation. The black cross lines indicate the principal stress directions and intensity. The red time labels denote the corresponding geological time intervals of the model. (a) In the early stage of collision (50 Ma), the entire model experiences a pronounced N-to NE-directed shortening. (b) As the continental convergence continues, the direction of the maximum principal stress experienced obvious rotation from the collision front zone to the area farther away. (c and d) During the main stage of the large-scale block extrusion (30–20 Ma), the regional stress field varies greatly due to block rotation and extrusion. (e and f) The modern tectonic framework has been established, with a stress field similar to those derived from the present GNSS data.

The most striking structural features in the SE Tibetan Plateau are a series of continental-scale shear zones (SZs), which extend for hundreds to more than a thousand kilometers (Fig. 1a). Our numerical models reconstruct the Cenozoic tectonic evolution of these major SZs (SZ1–8; Figs 3 and 4, Movie S1). In the early stage of continental collision (50 Ma), crustal shortening dominated the frontal convergence zone and the three weak zones (SZ1–3). It is noteworthy that these pre-existing structures are not defined as shear zones since the high strain is local, instead of whole strike, and no significant block displacement occurred at this stage (Fig. 3a). As the Indian Plate moved northward, a new shear zone (SZ4) developed to the east of the eastern indenter syntaxis, forming Block 5 (B5) bounded by SZ3 and SZ4 (Fig. 3b). Furthermore, strain was gradually concentrated along SZ5 to the northwest side of the strong South China Block (Fig. 3b). By 30 Ma, large-scale sinistral displacement occurred along SZ2 (Fig. 3c). SZ3 was also characterized by sinistral movement, especially in its southern segment, separating B6 from B5. However, its displacement was small compared with SZ2 and SZ4, adjusting the relative movement between B5 and B6 (Fig. 3c and d). Furthermore, several smaller conjugate shear zones formed within the northwest side of the original B3. A new continuous shear zone (SZ6) developed to the north of SZ4. Both B5 and B6 have experienced large-scale southeastward extrusion and clockwise rotation, which were mostly absorbed by the sinistral displacement along SZ2 and dextral shearing along SZ4 and SZ6 (Fig. 3d).

By ∼10 Ma, the large-scale block extrusion of B5 and B6 gradually ceased (Fig. 3e). Crustal deformation was characterized by development of new shear zones with clockwise rotation around the eastern indenter syntaxis (Fig. 3e). SZ7, which is characterized by sinistral movement, developed along the western margin of the South China Block (Fig. 3e). SZ1 was also reactivated and linked with SZ7 to form a new continuous sinistral shear zone. Together with SZ2 in the south, B7 developed with a rhomboidal shape. It is noteworthy that the rhomboidal B7 shows a southeastward slip with clockwise rotation. Such movement drove a transition in the motion of SZ2 from sinistral to dextral strike–slip (Fig. 3e and f). Furthermore, the NE-striking SZ8 formed adjacent to SZ5, separating B7 into two sub-blocks (Fig. 3e and f).

### Spatial–temporal variation of crustal deformation and rheology

At the early stage of collision (50 Ma), the entire model experiences pronounced N- to NE-directed shortening, with strain primarily localized along the weak zones (Fig. 4a). In the eastern oblique convergent zone, the regional stress field is predominantly NNE- to NE-directed. Additionally, the strain gradually weakens from south to north and from west to east. As the continental convergence continues, the direction of the maximum principal stress experienced clockwise rotation from the collision front zone to the area farther away (Fig. 4b). The extensional stresses also increase in magnitude in local areas such as in the western part of B3 and B4. During the main stage of the large-scale block extrusion (between 30 and 20 Ma), the displacement was mostly localized along the major shear zones with limited internal deformation within the blocks. The regional stress field varies greatly due to block rotation and extrusion (Fig. 4c and d). For example, the SE part of B2 shows a negative dilatation rate due to the obstruction of a rigid block to the east. The NW part of B6 shows obvious extensional deformation, possibly resulting from the SE block extrusion. Furthermore, previous negative dilatation rates along the pre-existing weak zones, such as SZ6 and SZ2, mostly change to positive values, indicating compression to extension (Fig. 4b and c). With the formation of the rhomboidal B7 at ∼10 Ma, the modern tectonic framework has been established. SZ7, SZ1 and SZ4, along with SZ6, absorbed most strain. Furthermore, the stress field within the extruded B5 and B6 is dominated by nearly E–W- to NW-directed extension.

**Figure 5.**
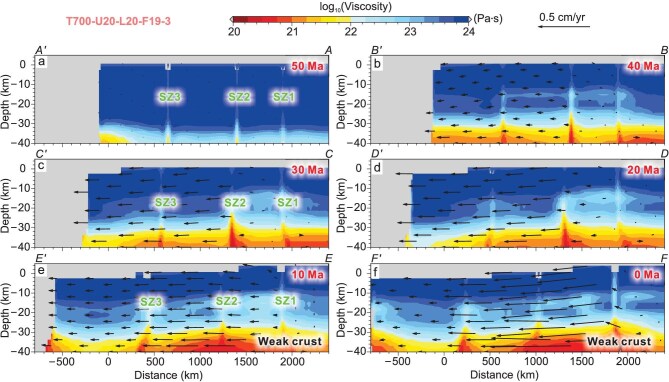
Profiles showing the evolution of crustal viscosity. The black arrows represent the projections of the velocities of the crustal materials in 3D space onto the plane of the cross-section. The locations of the cross-section are marked in Fig. 3. (a) At 50 Ma, the whole crust exhibits a quasiuniform rigid state with bulk viscosity exceeding 10^22^ Pa·s. (b) At ∼40 Ma, mid-lower crustal materials are rheologically weakened under higher temperature-pressure conditions. The shear zones show reduced viscosity compared to ambient crustal materials at equivalent depths. (c and d) During the block extrusion stage, depth-dependent viscosity stratification remains stable, and shear zones experience intensified rheological weakening. (e and f) More low-viscosity materials have accumulated in the high plateau, and deeper weakened crust exhibits accelerated mobility compared to the shallower crust.

Our modeling results also reveal the crustal rheological characteristics at various depths and their evolution due to strain accumulation and thermal effects during the plateau growth. During the initial stage (∼50 Ma), blocks predominantly maintain a quasiuniform rigid state, with bulk viscosity exceeding 10^22^ Pa·s, including weak zones (Fig. 5a). During the crustal shortening, mid–lower crustal materials are rheologically weakened and the viscosity below 30 km depth drops below 10^22^ Pa·s (Fig. 5b). Within the 30–10 km depth, although crustal strength shows limited weakening, viscosity values remain above 10^22^ Pa·s, retaining relatively rigid rheological properties. The upper crust (<10 km depth) preserves high- viscosity (∼10^24^ Pa·s, Fig. 5b). Notably, since more strain was localized along the weak zones with enhanced energy dissipation, these zones exhibit (i) markedly reduced viscosity compared to ambient crust at equivalent depths, and (ii) incipient upward material migration tendencies (Fig. 5b).

During the block extrusion stage, while depth-dependent viscosity stratification remains stable, shear zones (e.g., SZ2) experience intensified rheological weakening and upward material advection due to strain localization and dip–slip motion (Fig. 5c and d). At 10 Ma, more weakened crustal materials accumulated in the plateau interior, which would establish a flow gradient toward the plateau margin (Fig. 5e and f). Accordingly, deeper weakened crust exhibits accelerated mobility compared to the shallower crust, which could drive upper-crustal deformation (Figs S18–S20).

## DISCUSSION

### Cenozoic tectonic evolution of the SE Tibetan Plateau

A synthesis of field observations, geochronological analyses and geophysical investigations reveals a three-stage tectonic evolution across the SE Tibetan Plateau in the Cenozoic, including crustal shortening, lateral block extrusion and kinematic reversal [3,4]. Our numerical simulations successfully reconstruct the three distinct tectonic phases.

During the early Cenozoic (50–35 Ma), our model indicates that crustal deformation was dominated by NNE- to NE-directed shortening (Fig. 4a and b). This is consistent with Paleogene transpressional deformation identified from the Indochina Peninsula to Yunnan due to early oblique collision [3,9]. Anticline–syncline pairs and fold–thrust systems developed in the Meso–Cenozoic strata such as in the Jianchuan Basin, Lanping–Simao Basin, Chuxiong Basin, Khorat Plateau and Phuquoc–Kampot Som Basin, which indicate nearly E–W- to NE–SW-directed compression in the present coordinates [5,9,36] (Table S3). High-grade metamorphism, folds of gneiss/migmatite foliations at various scales, and anatexis evidenced by numerous leucocratic granitic dikes within metamorphic complexes or ductile shear zones such as the Ailaoshan–Red River, Gaoligong, Chongshan shear zones and Mogok, Doi Inthanon, Khanom and Stong metamorphic complexes, all point to crustal shortening and transpressional deformation [5–8]. Geochronological analyses, syntectonic sedimentary structures and magnetostratigraphy collectively constrain this shortening stage to the Eocene–Early Oligocene, with a general northward younging pattern [3,5,9,10]. Our modeling results align with the deformation patterns and paleostress reconstructions accounting for block rotations in the early Cenozoic.

Quasisynchronous initiation of major shear zones, such as the Gaoligong–Mogok, Chongshan–Lincang–Inthanon and Ailaoshan–Red River shear zones, accompanied by large-scale lateral block extrusion may have occurred since the early Oligocene in the SE Tibetan Plateau [3,4,8,37,38]. Petrological and structural analyses reveal mylonite and intense shear deformation fabrics characterized by steeply dipping foliations and subhorizontal mineral lineations along these shear zones [8,37–41]. Kinematic indicators such as asymmetric folds, boudinage, porphyroclast systems and S–C fabrics—coupled with geochronological data reveal: (i) right-lateral motion along the Gaoligong–Mogok shear zone during ∼32–17 Ma [7,40]; (ii) sinistral shearing along the Chongshan–Lincang–Inthanon shear and right-lateral tectonic overprinting near the EHS during ∼33–15 Ma [38,40,41]; (iii) left-lateral shearing along the Ailaoshan–Red River shear zone during ∼30–17 Ma [37,39,42]; and (iv) dextral movement along the Sagaing Fault in the Neogene [43], or earlier in the Oligocene along its western splays [44]. Notably, all shear zones exhibit general northward younging deformation/cooling ages [39,41,42].

Our model captures the formation of these shear zones (Fig. 3c). At ∼30 Ma, left-lateral motion along SZ2 (the Ailaoshan–Red River shear zone) and the right-lateral SZ4 (the Sagaing Fault or the Gaoligong–Mogok shear zone) control the extrusion and clockwise rotation of B5 (the Baoshan–Shan Block) and B6 (the Indochina–Simao Block). Left-lateral motion along the southern SZ3 (the Chongshan–Lincang–Inthanon shear zone) adjust the relative motion between B5 and B6. The newly formed SZ6 at SZ4’s northern terminus may represent the Jiali Fault, which is characterized by dextral movement (Fig. 3c and d). Apart from the Sagaing Fault still being active, most shear zones above ceased major motion by ∼15 Ma, generally aligning with field and geochronological observations.

A major kinematic reorganization occurred in the mid to late Miocene, establishing the modern tectonic framework of the SE Tibetan Plateau [3,4]. Key transitions included a switch from sinistral strike slips to dextral movement along the Red River Fault during 14–10 Ma [42], onset of the sinistral Xianshuihe–Xiaojiang Fault system at ∼12–10 Ma [45,46] and clockwise rotational velocity field around the EHS bounded by the Sagaing and Xianshuihe–Xiaojiang Faults with diffuse deformation in their interior domains [34]. Our results reproduce this transition process in the mid to late Miocene. First, the simulations reveal localized strain migration from the Ailaoshan–Red River shear zone (SZ2) to the sinistral Xianshuihe–Xiaojiang Fault system (SZ1 and SZ7), which delineates the northern and eastern boundary of the Chuandian rhombic Block (B7) (Fig. 3e). Second, the SZ8 development corresponds to the initiation of Lijiang–Xiaojinhe Fault, separating B7 into two sub-blocks. It is noteworthy that SZ8 formed along the SW extension of SZ5 (the Longmenshan Fault) in the late Miocene, and moved to its modern position due to the SE slip of B7 (the Chuandian Block) (Fig. 3e and f). Third, as the southeastward slip rate of the Chuandian Block (B7) exceeds that of the Indochina–Simao Block (B6), SZ2 (the Ailaoshan–Red River shear zone) switched to dextral motion.

Our model results show a crust motion trend similar to the present-day crustal velocity and stress–strain fields derived from the GNSS observations [47] (Fig. 1b and c). For example, the model shows a clear clockwise rotation around the indenting EHS, bounded by SZ4 (the Sagaing Fault), SZ1 and SZ7 (the Xianshuihe–Xiaojiang Fault), consistent with present-day GNSS observations (Fig. 3e and f). The Himalayan frontal zone is characterized by N–S-directed crustal shortening, which rotates to nearly NE–SW compression in the plateau interior (Figs 1c and 4f). Compressional domains are also identified along the Longmenshan Fault zone and adjacent regions (Figs 1c and 4f). Within the Chuandian Block, the GNSS-derived stress field shows that principal extension trends are nearly N–S in the north and rotate to nearly E–W/NE–SW in the south, especially near the Red River Fault and southern Xiaojiang Fault segments (Fig. 1c). In addition, the Tengchong Block is characterized by nearly E–W-directed extension. Except for the local regions in B7 (Chuandian Block) where the modeled principal stress deviates from the observed strain direction (Fig. 4f), other features, such as extension at the northern Red River Fault, Tengchong Block and throughout the Indochina Peninsula, are mostly reproduced (Fig. 4f).

### Crustal rheology and its impacts on deformation

Our model results suggest that crustal rheology profoundly affects the tectonic evolution and crustal deformation. In this section, we explore the evolutionary process of model viscosity, a core parameter that characterizes rheological properties, and its impact on strain partitioning under different boundary conditions. We set the initial blocks and weak zones with various rheological parameters. Such horizontal heterogeneity controls the formation and geometric distribution of major shear zones. Accordingly, the formation mechanisms of large-scale boundary faults can be summarized into two categories: (i) reactivation of pre-existing structural weak zones (e.g., SZ2, SZ3); and (ii) new faulting along boundaries between crustal units with significant differences in rheological properties (e.g., SZ5, SZ7).

Crust with diverse rheology also shows different deformation characteristics. Generally, blocks with relatively strong rheological properties (high-viscosity) are less deformed with strain mainly localized along their boundaries; on the contrary, blocks with weak rheological properties absorb more strain and yield a diffuse deformation. For example, we add a relatively rigid small block within the Chuandian Block to represent the Emeishan large igneous province (ELIP) (Figs S21 and S22). The results show that the strain along SZ8 and SZ7 is significantly enhanced due to the existence of the rigid ELIP (Fig. S21) and the slip direction of B7 is more inclined to the south compared with the reference model (Fig. S22).

Rheological heterogeneity also exists vertically within the crust. In this study, we tested a series of models with varying upper and lower crust thicknesses, showing that the upper–lower crust thickness discrepancy modulates the crustal deformation pattern. When the lower crust is relatively thick, strain is distributed across the abundant weakened lower crust, resulting in less strain concentrated along the shear zones in the upper crust (Fig. S3). In contrast, a thicker upper crust would lead to intense strain localization within the shear zone (Fig. S4), accompanied by a reduction in the weakening degree of the lower crust (with relatively high viscosity). It is worth emphasizing that although a thicker upper crust favors strain localization, the development of a mechanically weak middle–lower crustal layer is also a key prerequisite for large-scale shear zone formation [26].

Finally, the boundary temperature corresponding to the Moho depth also has a significant impact on the entire crustal rheological structure (Figs S1 and S2). It would alter the vertical rheological structure during crustal deformation, thereby affecting tectonic evolution and strain distribution. When the basement temperature is relatively low (e.g., 600°C), the rheological difference between the upper and lower crust is small. The whole crust behaves as a unified rigid block, and there is no significant velocity difference or decoupling between the upper and lower crust (Fig. S1). On the contrary, when the basement temperature is high (e.g., 700°C, 800°C, 900°C), the viscosity of the lower crust is significantly reduced, enhancing prominent lower crustal flow (Fig. S2).

### Geodynamic implications

Our simulation results not only quantitatively reconstruct the Cenozoic tectonic evolution of the SE Tibetan Plateau, but also offer new insights into the dynamic models during the plateau growth. The traditional tectonic escape model includes two-stage block extrusion: the Indochina Peninsula was first extruded southeast, bounded by the sinistral Ailaoshan–Red River and dextral Gaoligong–Mogok shear zones; and the second stage of the South China Block extrusion resulted in the kinematic shift of the Red River Fault [11,19]. However, this tectonic model fails to explain the formation of the Xianshuihe–Xiaojiang Fault and relatively diffuse crustal clockwise rotation around the EHS [11,19,37]. Our modeling results confirm the first-stage block extrusion with intense strain localization along boundary shear zones and minimal intra-block deformation (Fig. 3c). Furthermore, the dominant role of escape tectonics was likely replaced by crustal rotation, possibly accompanied by lower crustal flow since the mid–late Miocene. The later deformation stage is marked by the onset of the Xianshuihe–Xiaojiang Fault, slip reversal along the Red River Fault, formation of the Chuandian Block and its rotation around the EHS, and a transition from localized strain to relatively distributed deformation.

The lower crustal flow model proposed by Royden *et al.* [12] indicates that thermally activated, fluid-rich mid–lower crustal materials were generated beneath the plateau interior and likely propagated outward for hundreds of kilometers, driving crustal deformation [12,14,16]. Our numerical simulations show that lower crustal materials accumulated more in the high plateau and are characterized by higher velocities, which may have affected upper crustal motion fields through basal traction since the mid–late Miocene (Fig. 5e and f). However, our results indicate that most weak middle- to lower-crustal materials formed *in situ* or experienced relatively limited displacements (Fig. 5e and f). The differential motion between various crustal levels results from diverse strain responses of the dual-layered rheological architecture under a unified stress field. In addition, the low-viscosity crustal materials may have accumulated in the early stage of continental convergence (Fig. 5b).

Deeper dynamic processes may have also influenced regional lithospheric deformation. The removal of relatively cold, dense lower lithosphere due to convective instability and subsequent replacement with hotter material may have led to surface uplift and rapid river incision, quasisimultaneous deformation associated with plateau expansion, and potassic magmatism [48]. Tearing of the Indian subducted slab may have occurred beneath the Burmese Arc, resulting in upwelling of asthenospheric materials and clockwise toroidal mantle flow centered on the EHS [28,49]. However, this study mainly focuses on the crustal Cenozoic tectonic evolution, deformation pattern and evolving rheological characteristics, as well as their impacts. Therefore, our model cannot capture crust–mantle coupling and mantle dynamic processes (e.g., slab rollback, slab tearing, asthenospheric flow), which will be explored in future studies.

## CONCLUSIONS

Our 3D thermomechanical models reconstruct the Cenozoic tectonic evolution of the SE Tibetan Plateau including three key stages: early crustal shortening, lateral block extrusion, and subsequent regional kinematic reversal. We demonstrate that the block extrusion model and the lower crustal flow model are not competing models but represent sequential processes controlled by evolving rheology and boundary conditions under a unified tectonic framework. This tectonic transition explains both localized fault movement and diffuse deformation, consistent with geological, geophysical and GNSS data, which underscore the impact of crustal rheology. Our study provides new insights into the mechanisms of intracontinental deformation during plateau growth.

## MATERIALS AND METHODS

### Governing equations

The 3D parallel thermomechanical model code LaMEM [30] solves the conservation equations for momentum, mass and energy by employing a geometric multigrid method:


(1) \begin{eqnarray*}
\frac{{\partial {\tau }_{ij}}}{{\partial {x}_j}} - \frac{{\partial P}}{{\partial {x}_i}} + \rho {g}_i = 0,
\end{eqnarray*}

(2) \begin{eqnarray*}
\frac{{\partial {v}_i}}{{\partial {x}_i}} = 0,
\end{eqnarray*}

(3) \begin{eqnarray*}
\rho {C}_{\mathrm{p}}\left( {\frac{{\partial T}}{{\partial t}} + {v}_i\frac{{\partial T}}{{\partial {x}_i}}} \right) = \frac{\partial }{{\partial {x}_i}}\left( {k\frac{{\partial T}}{{\partial {x}_i}}} \right) + {H}_{\mathrm{r}} + {H}_{\mathrm{s}}.
\end{eqnarray*}

Here, *x_i_*(*i* = 1,2,3) denotes Cartesian coordinates, *τ_ij_* is the Cauchy stress deviator, *P* pressure, *ρ* density, *g_i_* gravity acceleration vector, *ν*_i_ velocity, *C*_p_ heat capacity, *T* temperature, *k* thermal conductivity, *H*_r_ radioactive heating, and ${H}_{\mathrm{s}} = {\tau }_{ij} \times ( {{{\mathop \varepsilon \limits^{{.}} }}_{ij} - \mathop \varepsilon\limits^{.}{}^{{\mathrm{el}}}_{ij}} ) $ is shear heating. As described below, $\mathop \varepsilon\limits^{.}{}^{{\mathrm{el}}}_{ij} $ is the elastic component of the strain rate.

The constitutive equation describes the relationship between the deviatoric stress and the strain rate (${\mathop \varepsilon \limits^{{.}} }_{ij}$) tensor:


(4) \begin{eqnarray*}
{\mathop \varepsilon \limits^{{.}} }_{ij} = {\tau }_{ij}/2{\eta }_{{\mathrm{eff}}},
\end{eqnarray*}

where *η*_eff_ is the effective viscosity and ${\mathop \varepsilon \limits^{{.}} }_{ij}$ is defined by:


(5) \begin{eqnarray*}
{\mathop \varepsilon \limits^{{.}} }_{ij} = \frac{1}{2}\left( {\frac{{\partial {v}_i}}{{\partial {x}_j}} + \frac{{\partial {v}_j}}{{\partial {x}_i}}} \right) - \frac{1}{3}\frac{{\partial {v}_k}}{{\partial {x}_k}}{\delta }_{ij}.
\end{eqnarray*}

In this study, we consider visco-elasto-plastic rheology of rocks. ${\mathop \varepsilon \limits^{{.}} }_{ij}$ contains elastic $\mathop \varepsilon\limits^{.}{}^{{\mathrm{el}}}_{ij} = \frac{1}{{2G}}\frac{{{\mathrm{D}}{\tau }_{ij}}}{{{\mathrm{D}}t}}$, viscous $\mathop \varepsilon\limits^{.}{}^{{\mathrm{vs}}}_{ij} =\mathop \varepsilon\limits^{.}{}^{{\mathrm{vs}}}_{\rm II} \, \frac{{{\tau }_{ij}}}{{{\tau }_{{\mathrm{II}}}}}$ and plastic $\mathop \varepsilon\limits^{.}{}^{{\mathrm{pl}}}_{ ij} =\mathop \varepsilon\limits^{.}{}^{{\mathrm{pl}}}_{\rm II} \, \frac{{{\tau }_{ij}}}{{{\tau }_{{\mathrm{II}}}}}$ components. Here, $\frac{{{\mathrm{D}}{\tau }_{ij}}}{{{\mathrm{D}}t}} = \frac{{\partial {\tau }_{ij}}}{{\partial t}} + {\tau }_{ik}{\omega }_{kj} - {\tau }_{kj}{\omega }_{ik}$ denotes the Jaumann objective stress rate with the spin tensor ${\omega }_{ij} = \frac{1}{2}( {\frac{{\partial {v}_i}}{{\partial {x}_j}} - \frac{{\partial {v}_j}}{{\partial {x}_i}}} )$. *G* is the elastic shear modulus and the subscript II represents the square root of the second invariant of associated tensor such as the deviatoric stress ${\tau }_{{\mathrm{II}}} = {( {\frac{1}{2}{\tau }_{ij}{\tau }_{ij}} )}^{1/2}$. Rocks fail when the second invariant of deviatoric stress is greater than the yield stress *τ*_y_. The second invariant of plastic strain rate ($\mathop \varepsilon\limits^{.}{}^{{\mathrm{pl}}}_{\rm II} = {\tau }_{\mathrm{y}}/2{\eta }_{{\mathrm{eff}}}$) is calculated via the Drucker–Prager yield criterion:


(6) \begin{eqnarray*}
{\tau }_{\mathrm{y}} = \sin \varphi P + \cos \varphi C.
\end{eqnarray*}

Here, *φ* and *C* are the frictional angle and cohesion, respectively. Rocks undergo elastic and viscous deformation when stress is smaller than the yield stress. The viscous rheology is computed as:


(7) \begin{eqnarray*}
{\eta }_{{\mathrm{vis}}} = 0.5{\left(\mathop \varepsilon\limits^{.}{}^{{\mathrm{vs}}}_{\rm II} \right)}^{\left( {1 - n} \right)/n}{B}_{\mathrm{n}}^{ - 1/n}{\mathrm{exp}} \left( {\frac{{E + PV}}{{nRT}}} \right),
\end{eqnarray*}

where *n*, ${B}_n$, *E, V* and *R* are experimentally determined flow law parameters, representing stress exponent, material constant, activation energy, activation volume and gas constant, respectively. To conclude, the effective viscosity can be computed via the standard quasiviscous expression:


(8) \begin{eqnarray*}
{\eta }_{{\mathrm{eff}}} = {\mathrm{min}} \left[ {{{\left( {\frac{1}{{G\Delta t}} + \frac{1}{{{\eta }_{{\mathrm{vis}}}}}} \right)}}^{ - 1},{\mathrm{\ }}\frac{{{\tau }_{\mathrm{y}}}}{{2{{\mathop \varepsilon \limits^{{.}} }}_{{\mathrm{II}}}}}} \right].
\end{eqnarray*}

## Supplementary Material

nwag118_Supplemental_Files
