# Supplementary material for: Decoding the southeastern Tibetan Plateau growth: a 3D numerical simulation of Cenozoic crustal deformation
Source: Natl Sci Rev. 2026 Feb 27;13(6):nwag118. doi: 10.1093/nsr/nwag118 (PMC13037706; doi:10.1093/nsr/nwag118)
Supplement: nwag118_Supplemental_Files [file nwag118_supplemental_files.zip › R_Supplementary Materials_NSR.pdf]

Supporting Information for

Decoding the SE Tibetan Plateau Growth: A 3D Numerical Simulation of Cenozoic Crustal Deformation

Yuyang Wang[1], Yang Wang[1]*, Jianfeng Yang[2], Lijun Liu[2] Jinjiang Zhang[3] and Peizhen Zhang[1]

[1]Guangdong Provincial Key Laboratory of Geodynamics and Geohazards, School of Earth Sciences and Engineering, Sun Yat-Sen University, Guangzhou, 510275, China.

[2]State Key Laboratory of Lithospheric Evolution, Institute of Geology and Geophysics, Chinese Academy of Sciences, Beijing, China.

[3]The Key Laboratory of Orogenic Belts and Crustal Evolution, School of Earth and Space Sciences, Peking University, Beijing, 100871, China.

*Corresponding author: Yang Wang

Email: wangyang26@ mail.sysu.edu.cn

This PDF file includes:

Supporting text

Figures S1 to S23

Tables S1 to S3

Legends for Movies S1

References

Other supporting materials for this manuscript include the following:

Movies S1

SUPPORTING INFORMATION TEXT

Numerical technique

The numerical solutions of Stokes equations are obtained through the implementation of the Flexible Generalized Minimal Residual (FGMRES) Krylov subspace method(Saad, 1993), complemented by a geometric multigrid preconditioning strategy that concurrently optimizes pressure and velocity fields within the PETSc (https://petsc.org/release/) computational framework. This architecture leverages PETSc's parallelized linear algebra infrastructure for distributed matrix-vector operations, preconditioner construction, and nonlinear solver implementations. Our parallelization paradigm in LaMEM employs spatial decomposition of structured finite difference grids through distributed memory objects. Computational executions were conducted on the BSCC-T platform at Beijing Super Cloud Computing Center (https://cloud.blsc.cn/), with individual simulations utilizing 180 CPU cores distributed across three computing nodes. From an extensive suite of 83 systematically parameterized models, we present only those demonstrating optimal solution characteristics. Each large-scale simulation resolving shear zone dynamics (144×160×16 cells) required 1,000-3,000 timesteps, with typical runtimes spanning ~24 hours per model.

The multigrid preconditioning hierarchy incorporates four refinement levels, employing halved cell dimensions at successive coarser scales, ultimately transitioning to direct solvers (SuperLU_DIST or MUMPS) at the coarsest resolution. Nonlinear solution strategies employ adaptive iteration switching between Picard and Newton-Raphson methods, with the latter activated upon achieving a “good” residual to accelerate convergence. Solution convergence criteria are governed by absolute and relative tolerances of 10^{-3} and 10^{-4} respectively across 10-20 SNES iterations, incorporating the Eisenstat-Walker adaptive tolerance algorithm. Linear systems are addressed through the FGMRES Krylov method, constrained by dual tolerance thresholds of 10^{-8} within 15-30 iterations. Due to the inherent numerical challenges of elastoplastic deformation, convergence optimization frequently necessitates adjustments to multigrid parameters, augmented iteration limits, and line search algorithm tuning. The implemented free surface stabilization protocol(Kaus et al., 2010) synergizes with visco-elasto-plastic formulations,

which demonstrate enhanced strain localization fidelity(Jaquet et al., 2016) and improved numerical robustness(Duretz et al., 2020). This comprehensive approach ensures rigorous preservation of physical conservation laws while maintaining computational efficiency across multiphase rheological regimes.

Boundary condition testing

We conducted detailed sensitivity tests on these parameters and discussed the impacts of each boundary condition on the model individually. All the model cross-sections presented below are selected from the same location as that of the recommended model.

First, the boundary condition that exerts the most significant influence on the model's rheological parameters is the boundary temperature at Moho depth. Although extensive geothermal studies have indicated that the Moho temperature in the southeastern margin of the Tibetan Plateau exceeds 600 °C (Čermák, 1982; Clark et al., 2011; Schutt et al., 2018; Sun et al., 2022), its absolute value remains highly controversial. Therefore, we tested the basal boundary temperatures of 600, 700, and 800 °C as potential temperature boundary conditions, respectively. It can be clearly observed that the influence of boundary temperature is mainly concentrated on the rheological properties of the lower crust, with negligible effects on the viscosity of the upper crust (Figs. S1-S2, S7A-B, S8A-B). Generally, a low boundary temperature reduces the differences in rheological properties between the upper and lower crusts, leading to dominant rigid-block motion of the entire crust without significant velocity differences or decoupling between the upper and lower crusts (Figs. S1, S7A, S8A). In contrast, higher Moho temperature rapidly reduces the viscosity of lower crustal materials, resulting in a substantial increase in their fluidity and faster flow velocities, which causes premature decoupling from the upper crust (Figs. S2, S7B, S8B).

Similarly, under the condition of a fixed boundary temperature, the thickness of upper and lower crusts is also becoming a critical factor controlling the variations in their rheological properties. The thickness discrepancy between the upper and lower crust is primarily manifested in modifying the strain partitioning pattern. In models with a thicker lower crust, most of the strain is absorbed by the weakened lower crustal materials, leading to a relatively diffuse strain distribution throughout the entire model and an absence of

significant strain localization in the upper crust, particularly within the shear zones (Figs. S3, S7C, S8D). In contrast, the strain partitioning pattern differs remarkably in models with a thicker upper crust: strain is localized within the shear zones. The lower crust exhibits a low degree of weakening. (Figs. S4, S7D, S8D).

What's more, the material rheological properties within the free space also exert a certain influence on block deformation. However, this influence does not significantly alter the rheological properties of the blocks, but only slightly affects the magnitude and position of block motion. Such setup is mainly to simulate the trench retreat along the Sumatra-Java subduction zone. And it is only a secondary influencing factor in the model (Figs. S5-S6, S7E-F, S8E-F). Admittedly, the free space and weak zone geometries set in the model is simplified in order to improve computational efficiency. Such designed model references some classical physical simulation such as Tapponnier et al. (1982, 2001).

We set convergence rate as a constant value for two reasons. First, our simulations mainly focus the crustal evolution and deformation since 50 Ma. Based on previous studies, although the convergence rate of the Indian Plate has changed through time, it remained relatively stable at approximately 5 cm/yr around the ~47 Ma (Fig. S9). The minor initial variations during 50-47 Ma would not exert a substantial impact on the model runs. Second, the rates derived from paleomagnetic data are inherently associated with considerable uncertainties. A time-varying (dynamic) convergence rate would lead to an excessively large computational load for the model. While a constant value is the preferred choice for model fitting, we have still tested various models under different convergence rates. The detailed test parameters are presented in the table below.

As we can see, the convergence rates primarily govern the deformation duration and magnitude of the model. A relatively low convergence rate results in a significant delay in shear zone development, slower block motion velocities, and impeded strain localization within shear zones (Figs. S10-S11). In contrast, an excessively high convergence rate leads to a marked advance in shear zone initiation, accompanied by enhanced block deformation, faster block motion velocities, and a higher degree of strain localization within shear zones (Figs. S12-S13). The northern boundary of our model corresponds to the northern margin of the Songpan-Ganzi Black. The present-day GNSS-derived velocity in this region in the

stable reference frame of the Eurasian continent is approximately 1 cm/yr, whereas the indentation rate of the Indian Plate is around 4 cm/yr. Therefore, we set the convergence rate to 3 cm/yr in our model. What's more, the recommended model using 3cm/yr yield the results which are consistent with most existing geological and geophysical observations.

Quantitative comparisons

We have performed coordinate transformation and showed the velocity field representing the present-day state in our model, and then compared them with the velocity field derived from GNSS observations. We have projected two commonly used velocity fields, including the Eurasian reference frame and the South China reference frame (Fig. S14). The velocity field comparison shows that the overall motion trends of the Sichuan-Yunnan Block, the northeastern margin of the Tibetan Plateau, and the Eastern Himalayan Syntaxis are mostly consistent. Admittedly, there is a certain deviation in the velocity direction, which may be attributed to the following four key factors: 1. Temporal scale discrepancy: our modeled modern crustal velocity field is actually an average result over 1 Myr. 2. Simplification of complex geological units and processes: our model adopts significant simplifications for complex geological units and convergence processes. For example, the deviation in the velocity direction in the Himalayan region is due to the simplification of geometry of the Indian Plate and its indentation process in the model (Fig. S14). 3. Computational resolution constraints: Owing to the limitations of computational resources, it is impractical to achieve kilometer-level spatial resolution in our large-scale model. 4. Finally, it should be noted that the coordinate system adopted in our model does not strictly correspond to the actual latitude and longitude coordinates, and can only be roughly converted based on the model boundary extent. That's why the modeled velocities and strains rates are relatively smaller than the observed data.

What's more, we also calculated the slip rates of major faults, which are average value during the last 1 Myr. The model results show that the left-lateral slip rate of its northern segment is approximately ~10 mm/a (Fig. S14), which is consistent with existing observations. The Ganzi-Yushu fault (NW segment) shows a horizontal slip at a rate of ~13.4-14.3 mm/yr (Wang et al., 2008; Xu et al., 2003); while the Xianshuihe fault has recorded slip rates of ~9-12 mm/yr (Zhang et al., 2025 and references therein). The

Anninghe-Zemuhe fault (middle segment) accommodates a slip rate of ~6.5-7.3 mm/yr; the Daliangshan fault to the east show a left-lateral fault slip rate of ~3-4 mm/yr (He et al., 2008). Our model suggests 15 mm/yr along this fault segment. Geodetic and other relevant geological data indicate a Holocene fault slip rate of ~8.0-12.0 mm/yr of the Xiaojiang fault (Zhang et al., 2025 and references therein), which is consistent with our model results (10 mm/yr along the Xiaojiang fault;).The model results indicate the dextral slip rate of the Red River fault is ~5 mm/a (Fig. S14), which is also slightly higher than the current right-lateral slip rate of 1–4 mm/a for the Red River Fault (Shi et al., 2018; Li Z et al., 2020; Huang et al., 2023). To the west, the strain zone corresponding to the Sagaing Fault shows a slip dextral slip rate ranging from 20–25 mm/a (Fig. S14), which is consistent with the current right-lateral slip rate of ~20 mm/a for the Sagaing Fault (Simons et al., 2007; Mallick et al., 2019; Panda et al., 2020; Panda & Kundu, 2022).

The modeled strain rate field show two high-strain zones: the Xianshuihe–Xiaojiang Fault zone and the Sagaing Fault zone, which is consistent with the observations However, their strain rates in this model are systematically underestimated, as we mentioned before (Fig. 15).

Finally, we selected three representative topographic profiles from the DEM and extracted the topographic variations at the corresponding locations in the model for comparison.

The topographic profile along the southeastern margin of the Tibetan Plateau (A–B) and its corresponding counterpart in the model. In the profile across the SE Tibetan Plateau (A'–B'), the topography generally exhibits a gradual lowering trend over a distance of several hundred kilometers, with an elevation difference of approximately 4 km between the highest and lowest points, indicating a good consistency between the two (Fig. S16).

The topographic profile across the Longmenshan thrust (C'–D') shows a sharp topographic variation over a short distance. This type of abrupt topographic variation is also observed in the Himalayan profile (E–F), and the model well captures this trend (E'–F'). The absolute elevation is higher than the actual elevation. The reason is that we did not set an erosion rate in the model to preserve the tectonic information as much as possible. If the surface erosion is taken into account, the absolute topographic elevation would be much closer to the actual terrain (Fig. S16).

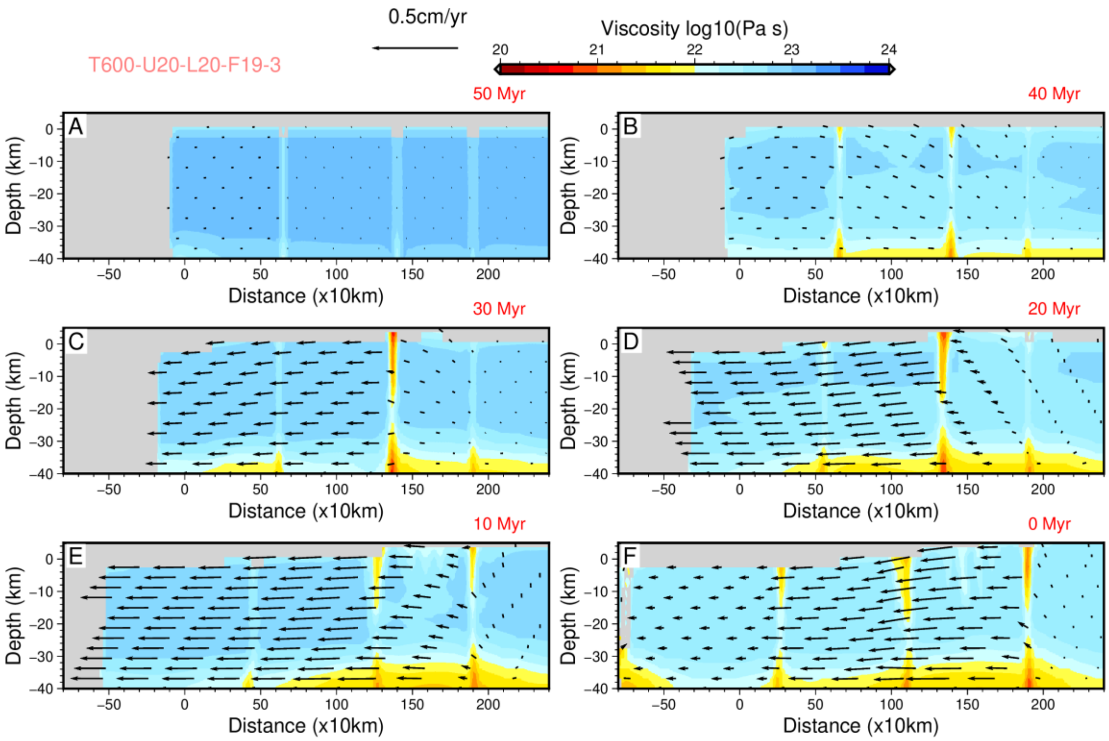

Figure S1 The evolution of six viscosity profiles in the model T600-U20-L20-F19-3, The black arrows represent the projections of the velocities of the materials in three-dimensional space onto the plane of the cross-section.

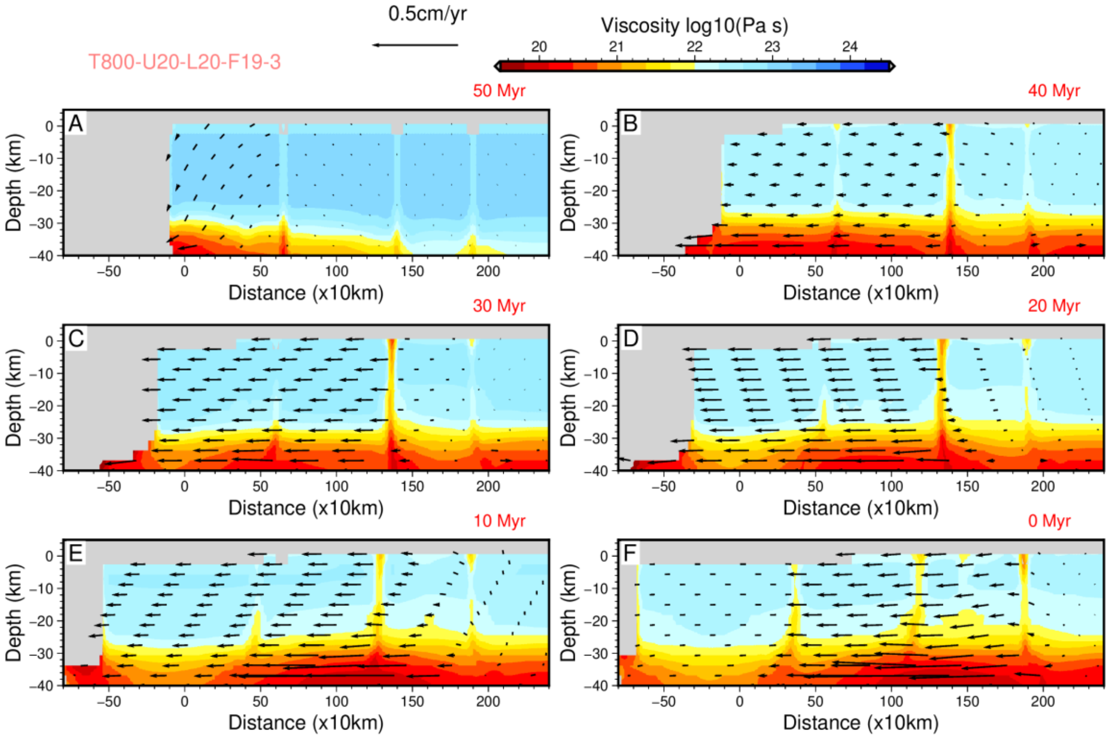

Figure S2 The evolution of six viscosity profiles in the model T800-U20-L20-F19-3, The black arrows represent the projections of the velocities of the materials in three-dimensional space onto the plane of the cross-section.

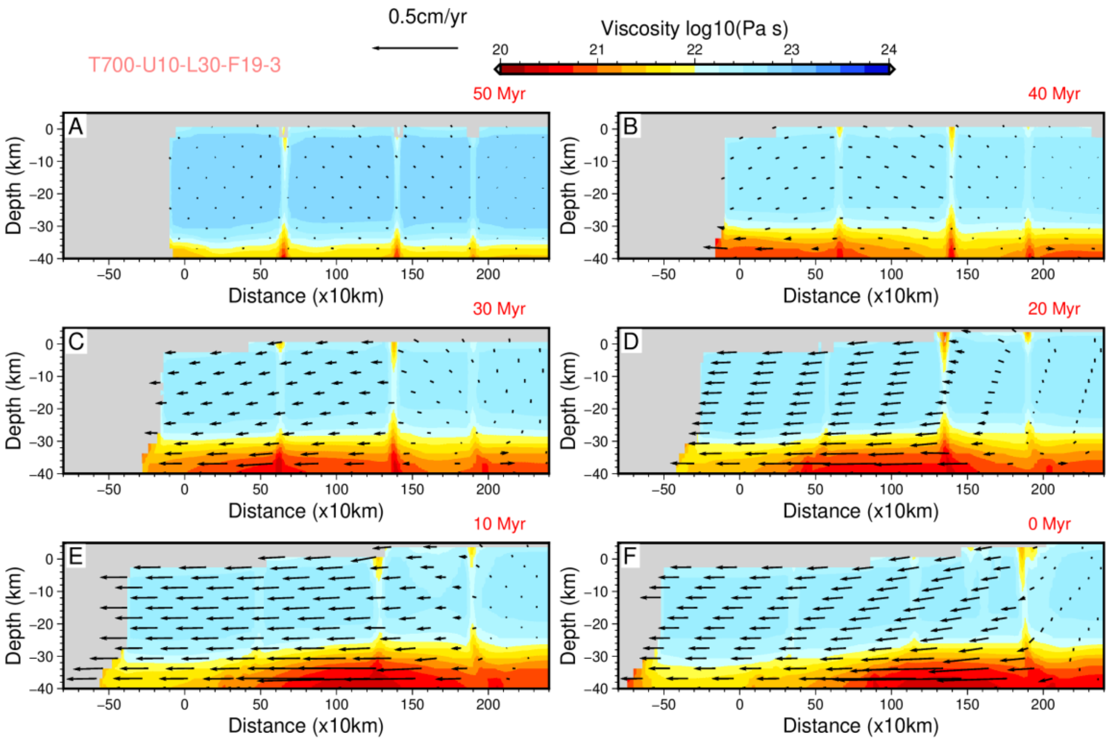

Figure S3 The evolution of six viscosity profiles in the model T700-U10-L30-F19-3, The black arrows represent the projections of the velocities of the materials in three-dimensional space onto the plane of the cross-section.

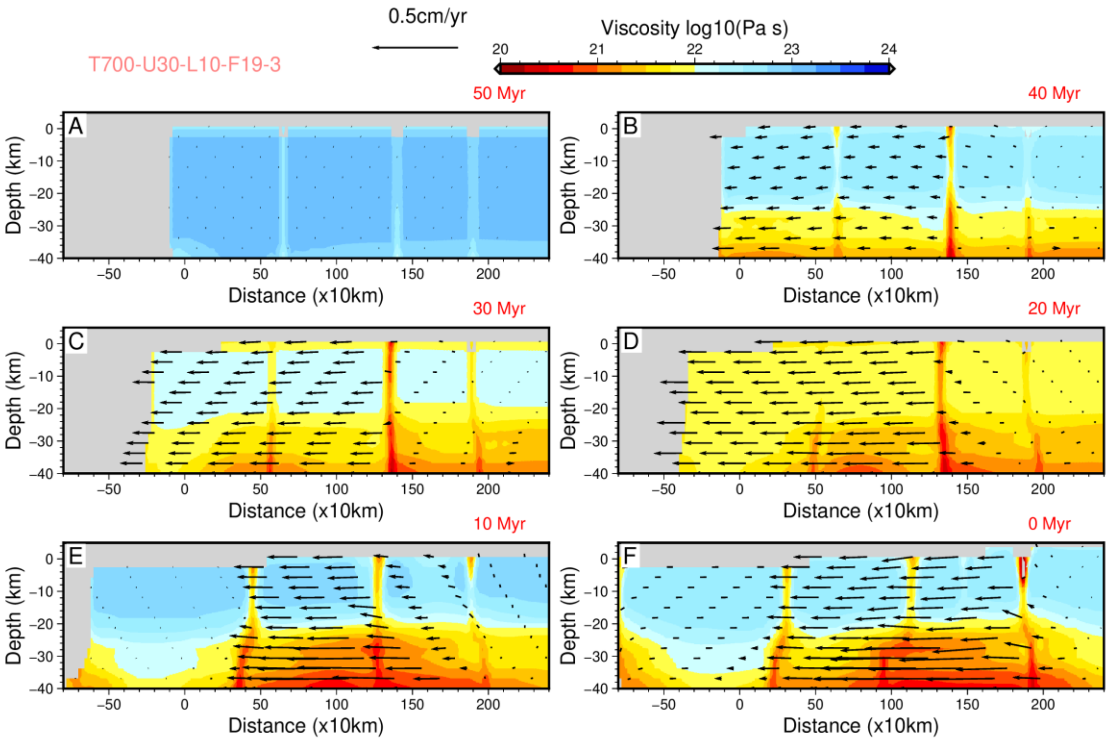

Figure S4 The evolution of six viscosity profiles in the model T700-U30-L10-F19-3, The black arrows represent the projections of the velocities of the materials in three-dimensional space onto the plane of the cross-section.

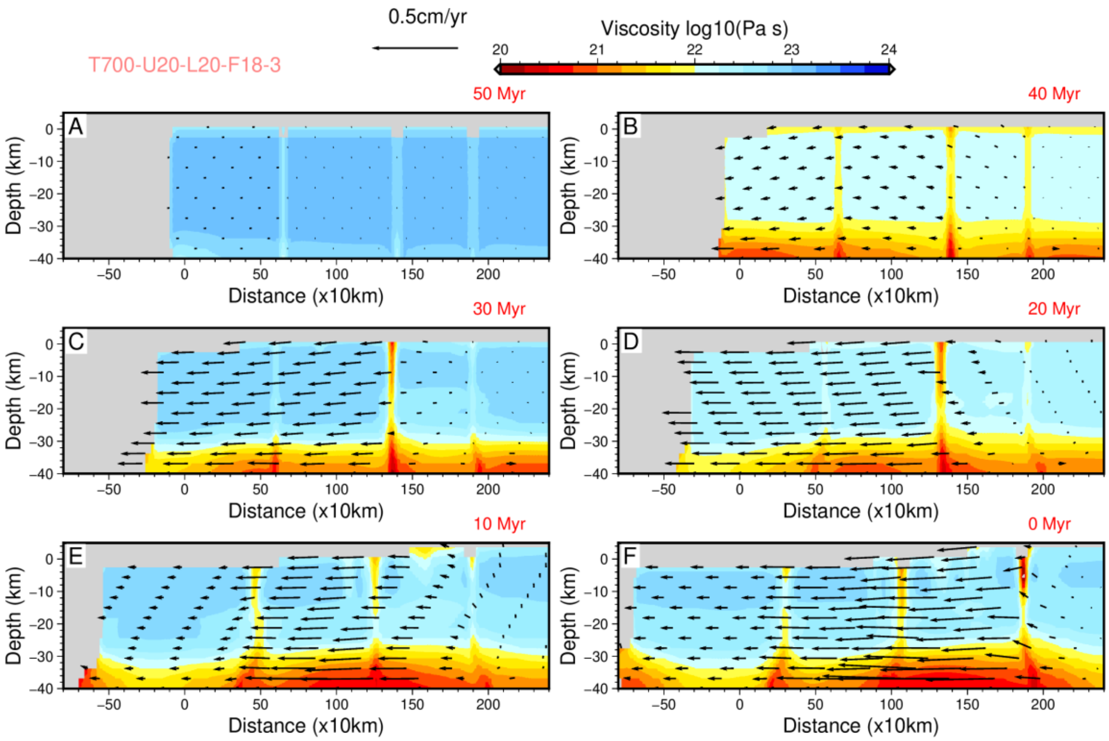

Figure S5 The evolution of six viscosity profiles in the model T700-U20-L20-F18-3, The black arrows represent the projections of the velocities of the materials in three-dimensional space onto the plane of the cross-section.

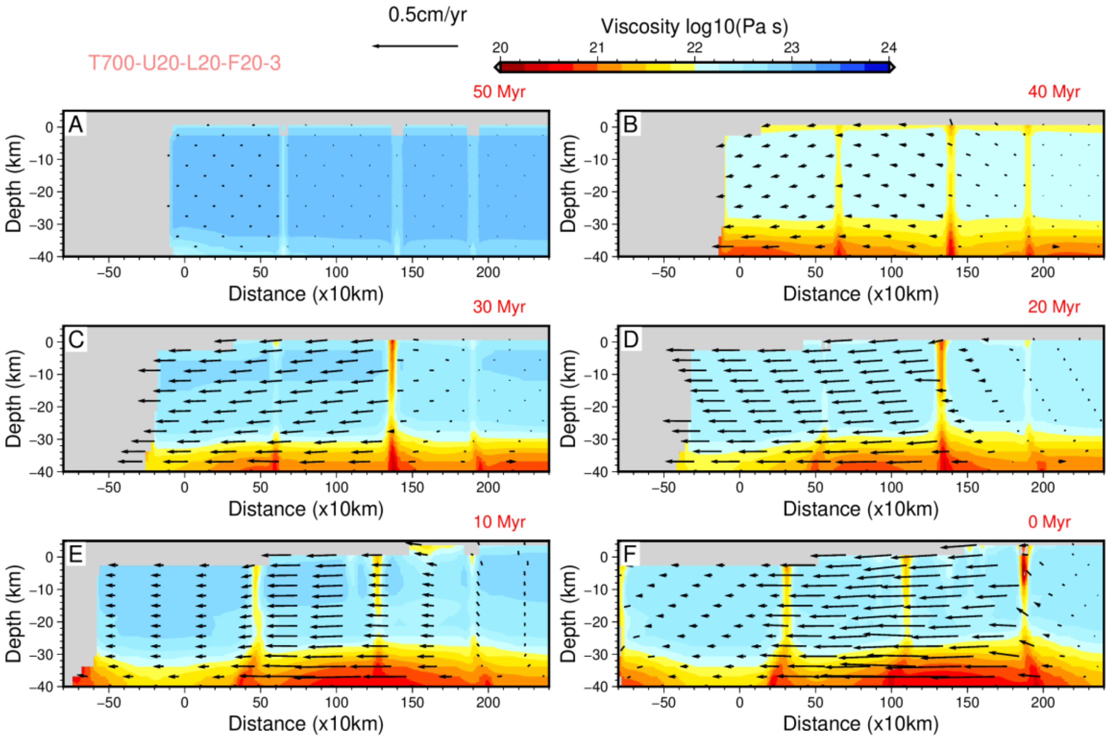

Figure S6 The evolution of six viscosity profiles in the model T700-U20-L20-F20-3, The black arrows represent the projections of the velocities of the materials in three-dimensional space onto the plane of the cross-section.

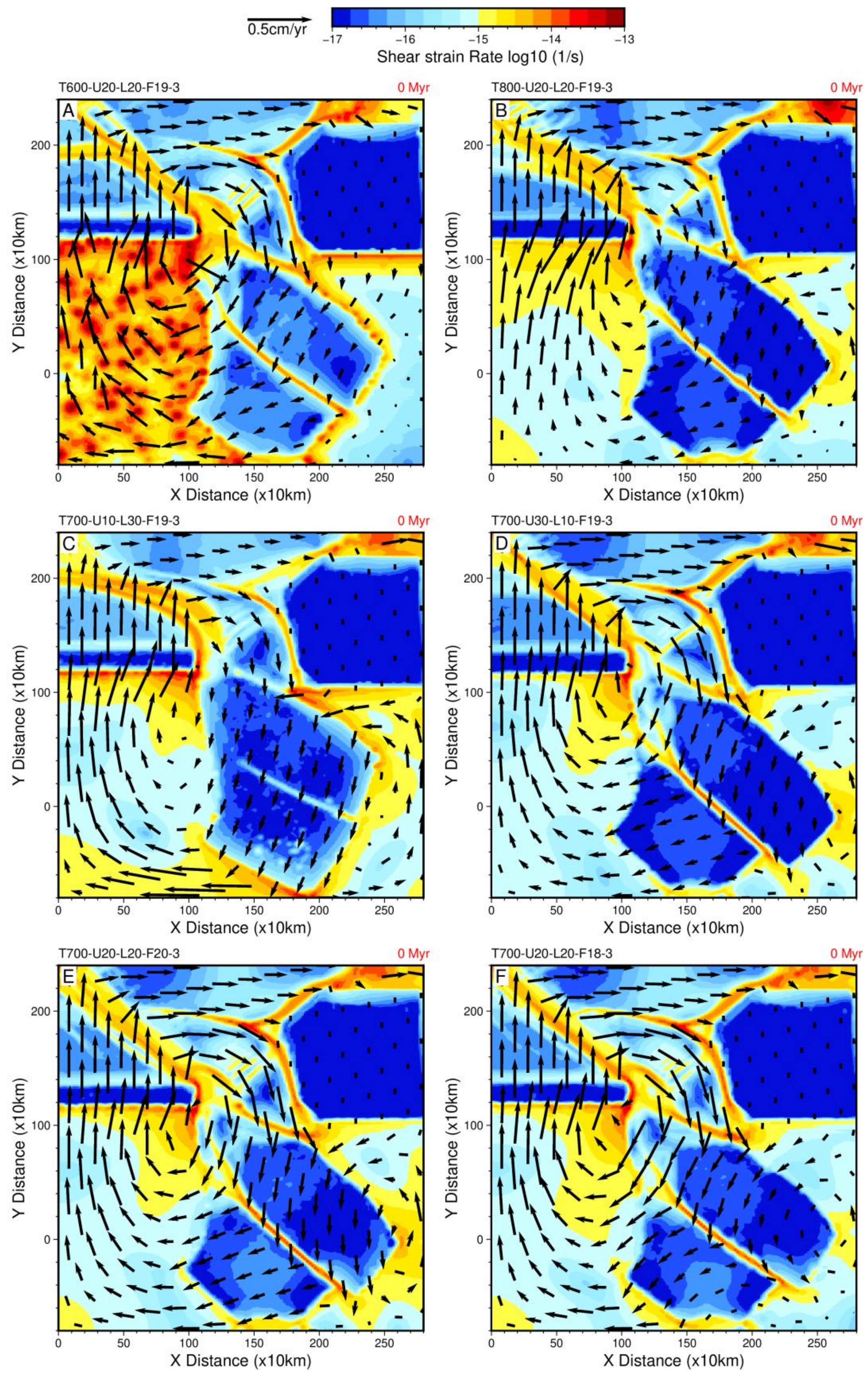

Figure S7 The shear strain rate with crustal velocity field of six models at 0 Ma.

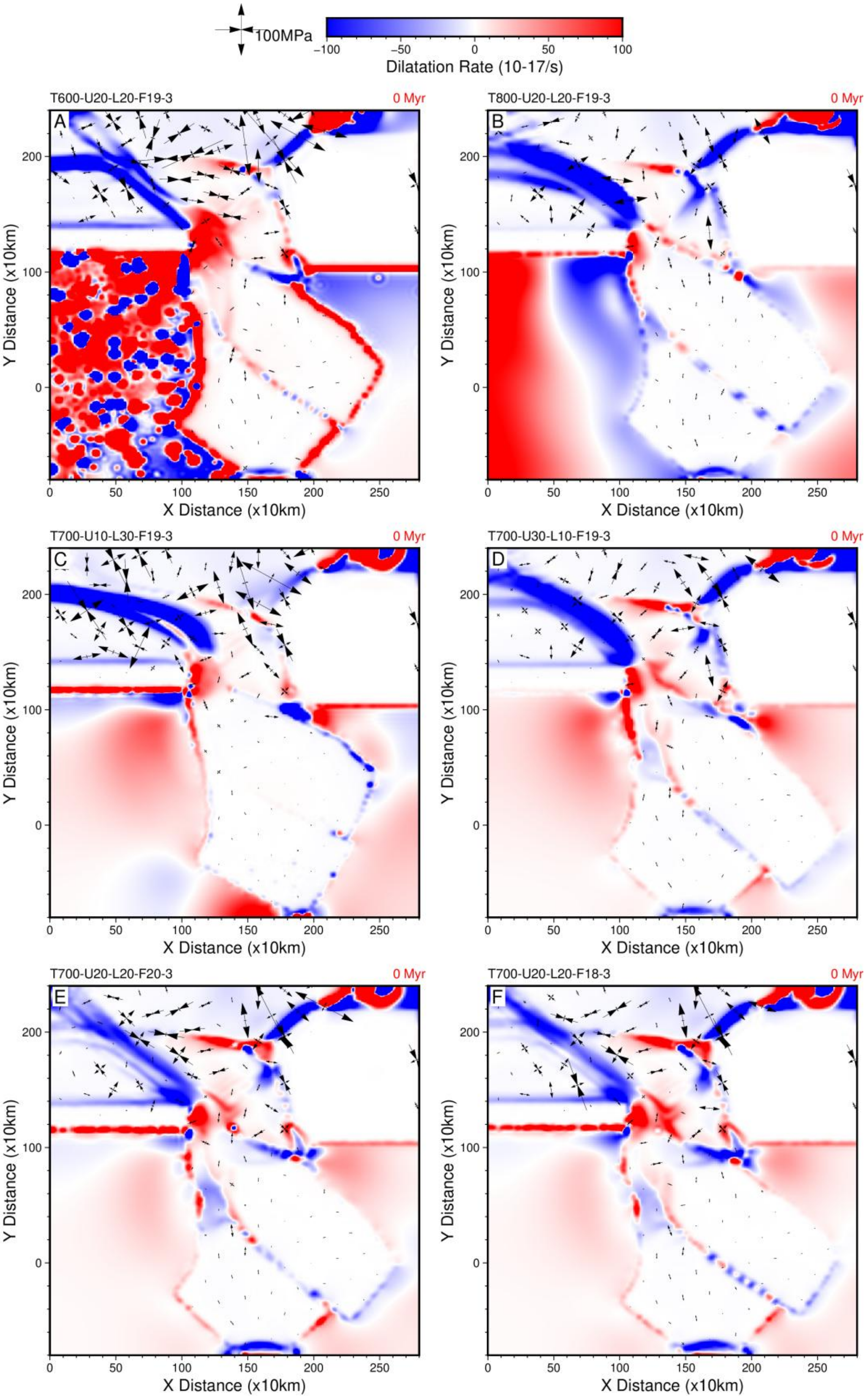

Figure S8 The dilatation rate of six models at 0 Ma.

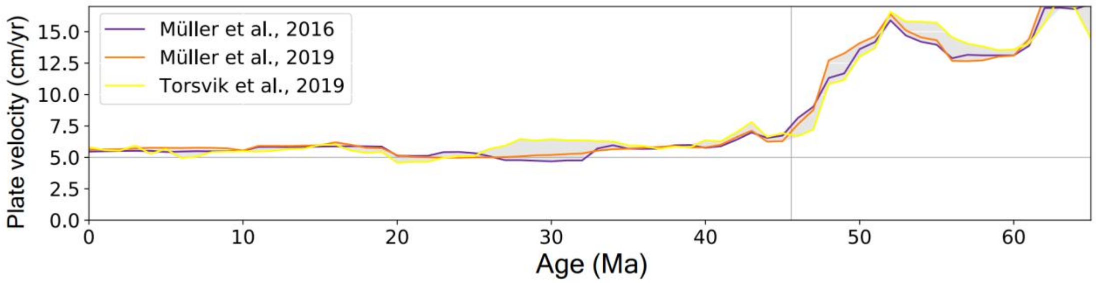

Figure S9 Convergence Rate Between the Indian and Eurasian Plates Derived from Paleomagnetic Data.

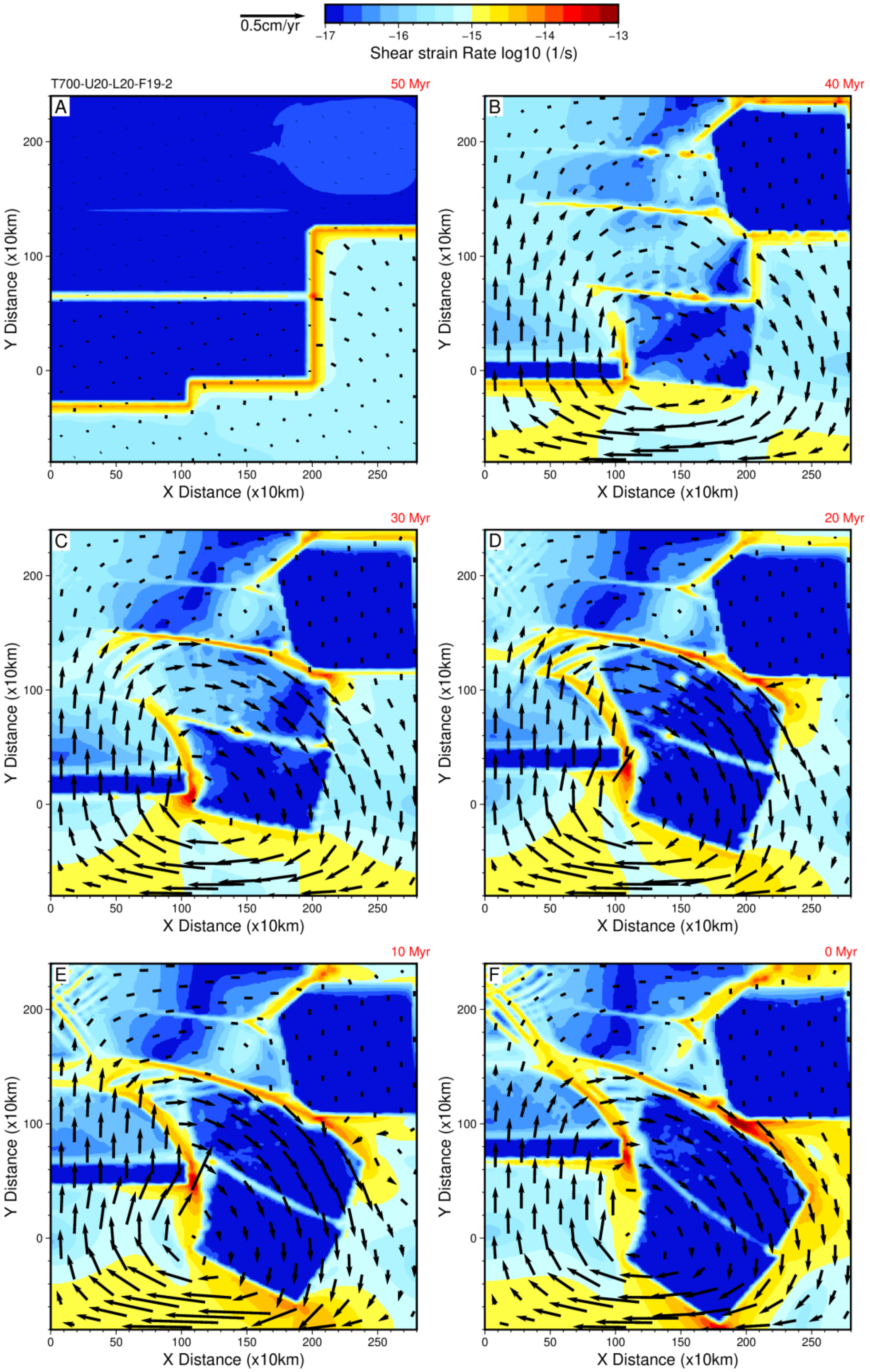

Figure S10 The evolution of shear strain rate with crustal velocity field of model T700-U20-L20-2.

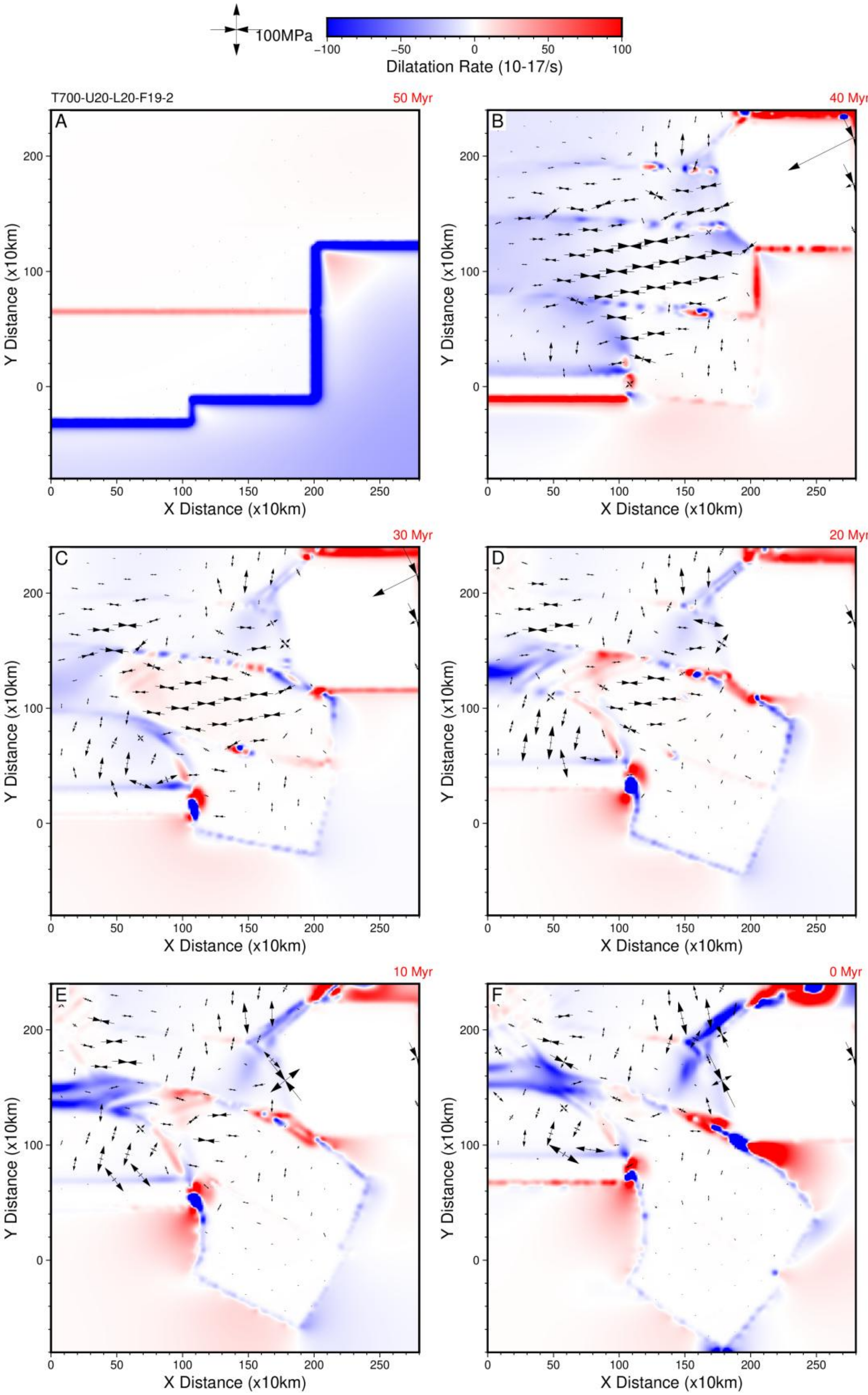

Figure S11 The evolution of dilatation rate of model T700-U20-L20-2.

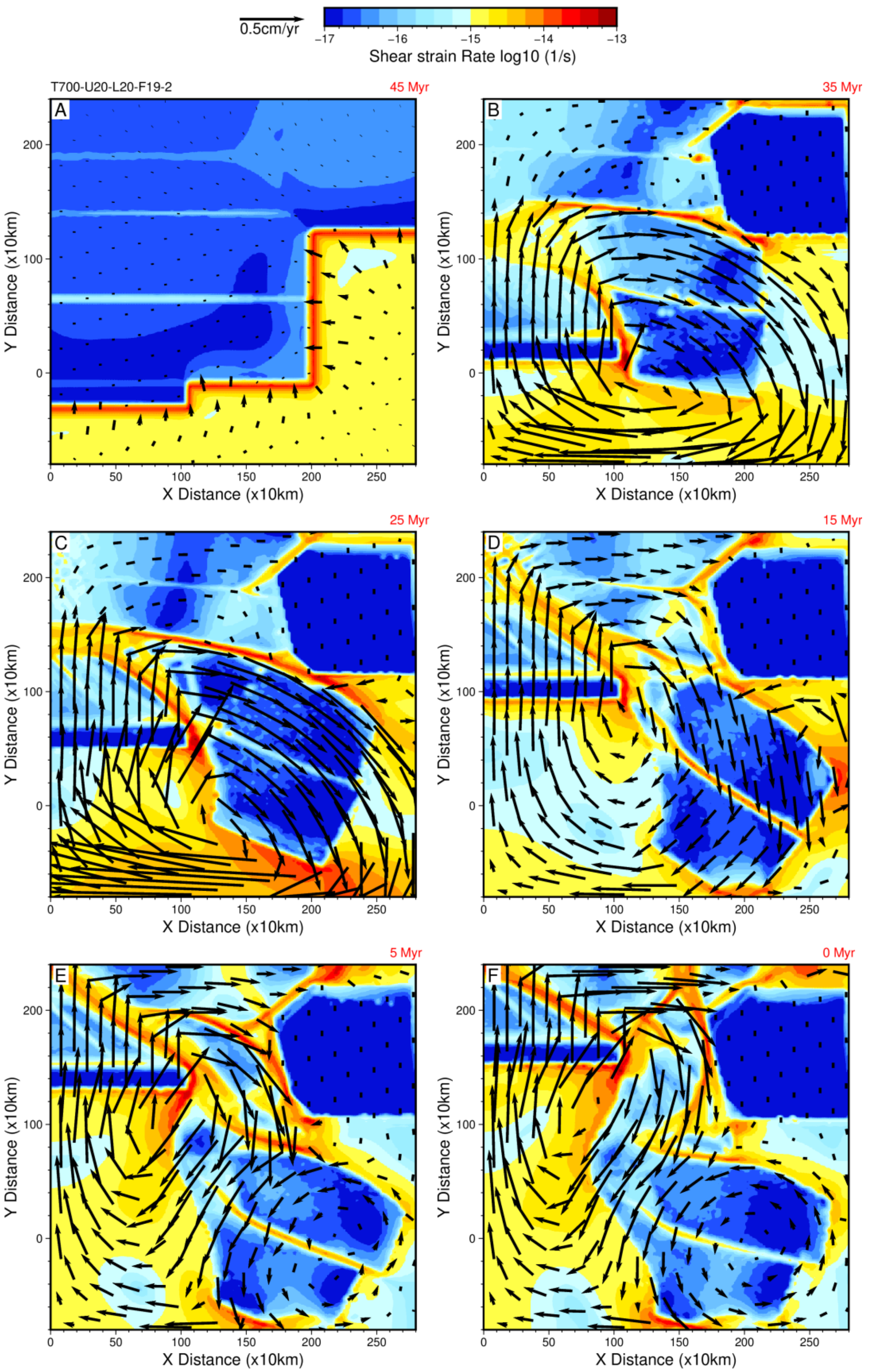

Figure S12 The evolution of shear strain rate with crustal velocity field of model T700-U20-L20-4.

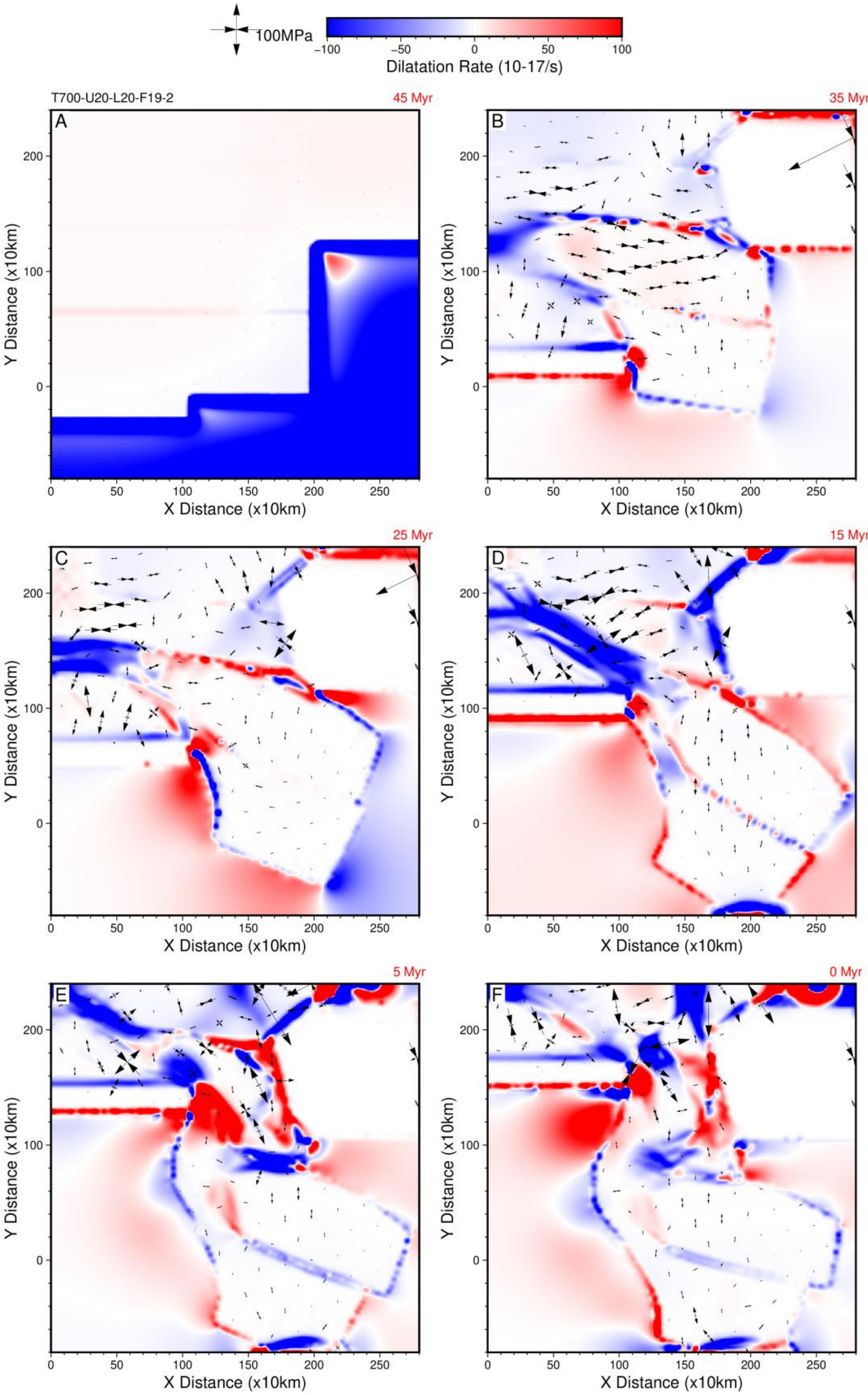

Figure S13 The evolution of dilatation rate of model T700-U20-L20-4.

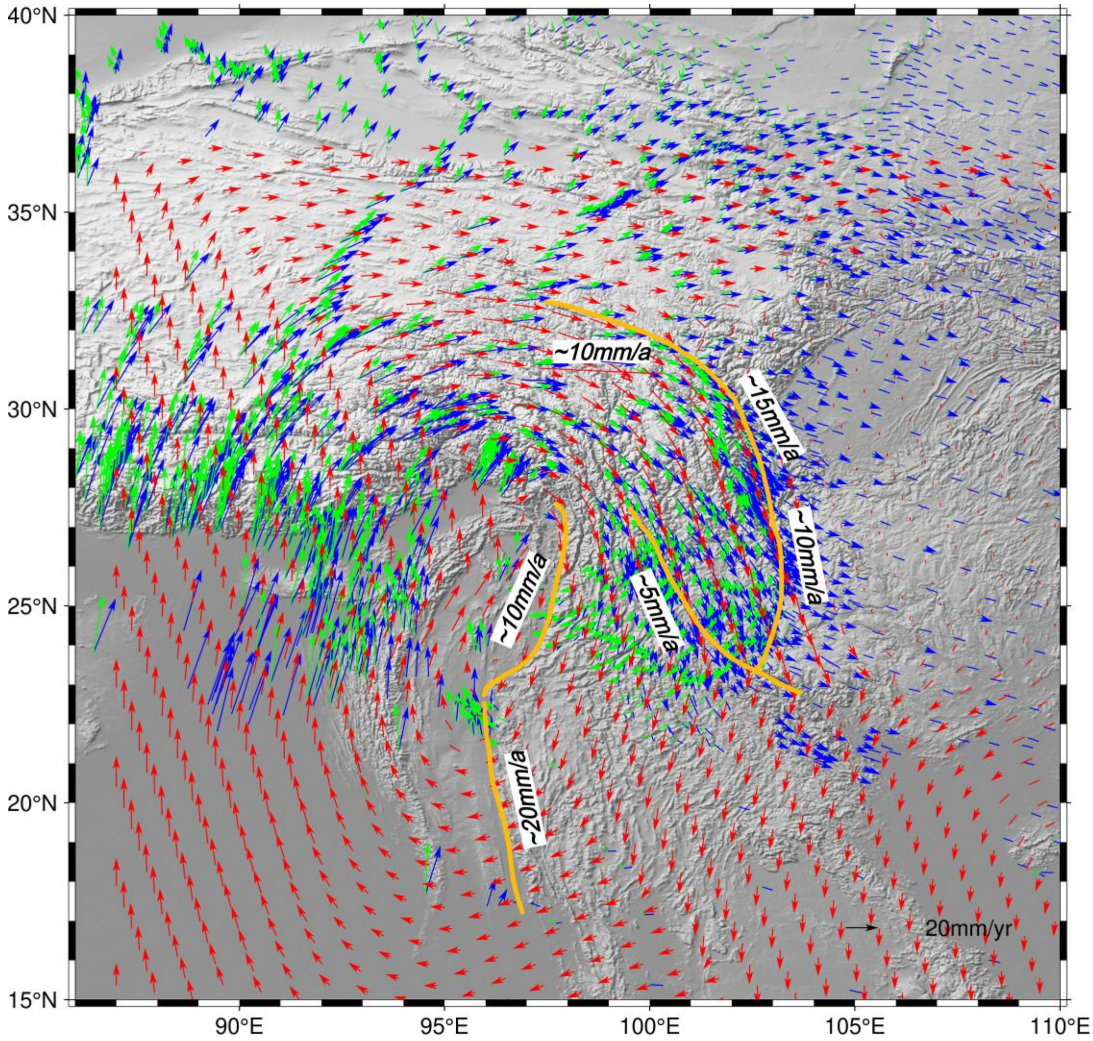

Figure S14 Comparison of GNSS velocity fields. Red vectors denote the velocity values predicted by the model (with coordinates roughly converted according to the model boundaries). Blue vectors represent the observed GNSS velocity data in the stable reference frame of the Eurasian continent(after Li et al., 2025). Green vectors represent the observed GNSS velocity data in the stable reference frame of the South China Block(after Xu et al., 2022). The fault slip rates were derived from the velocity differences between distinct model blocks.

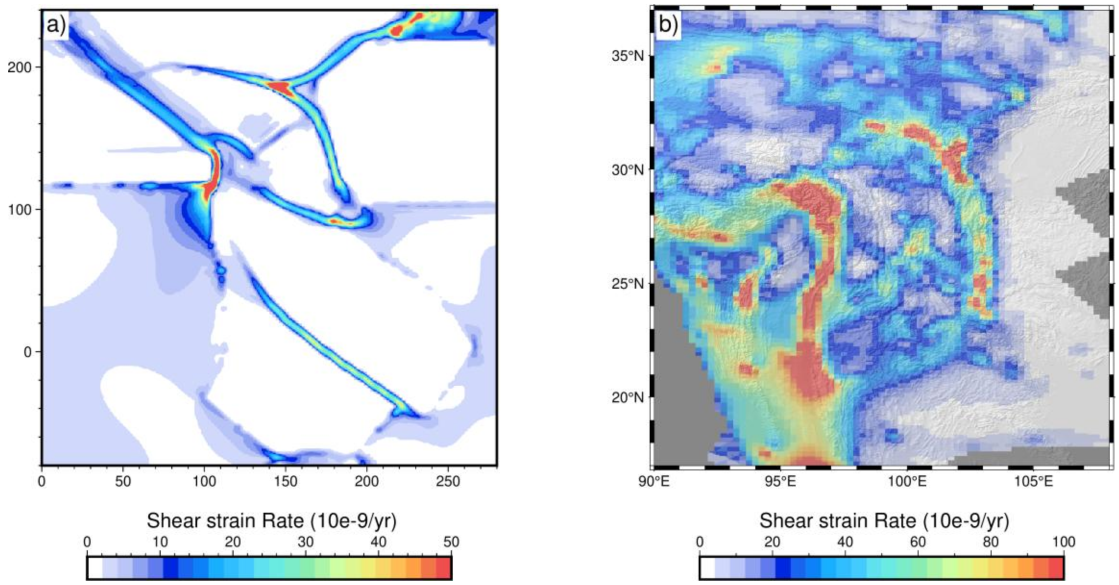

Figure S15 Comparison of shear strain rates in a unified unit. (a) Shear strain rates derived from the reference model; (b) Shear strain rates calculated from GNSS data (after Kreemer et al., 2014).

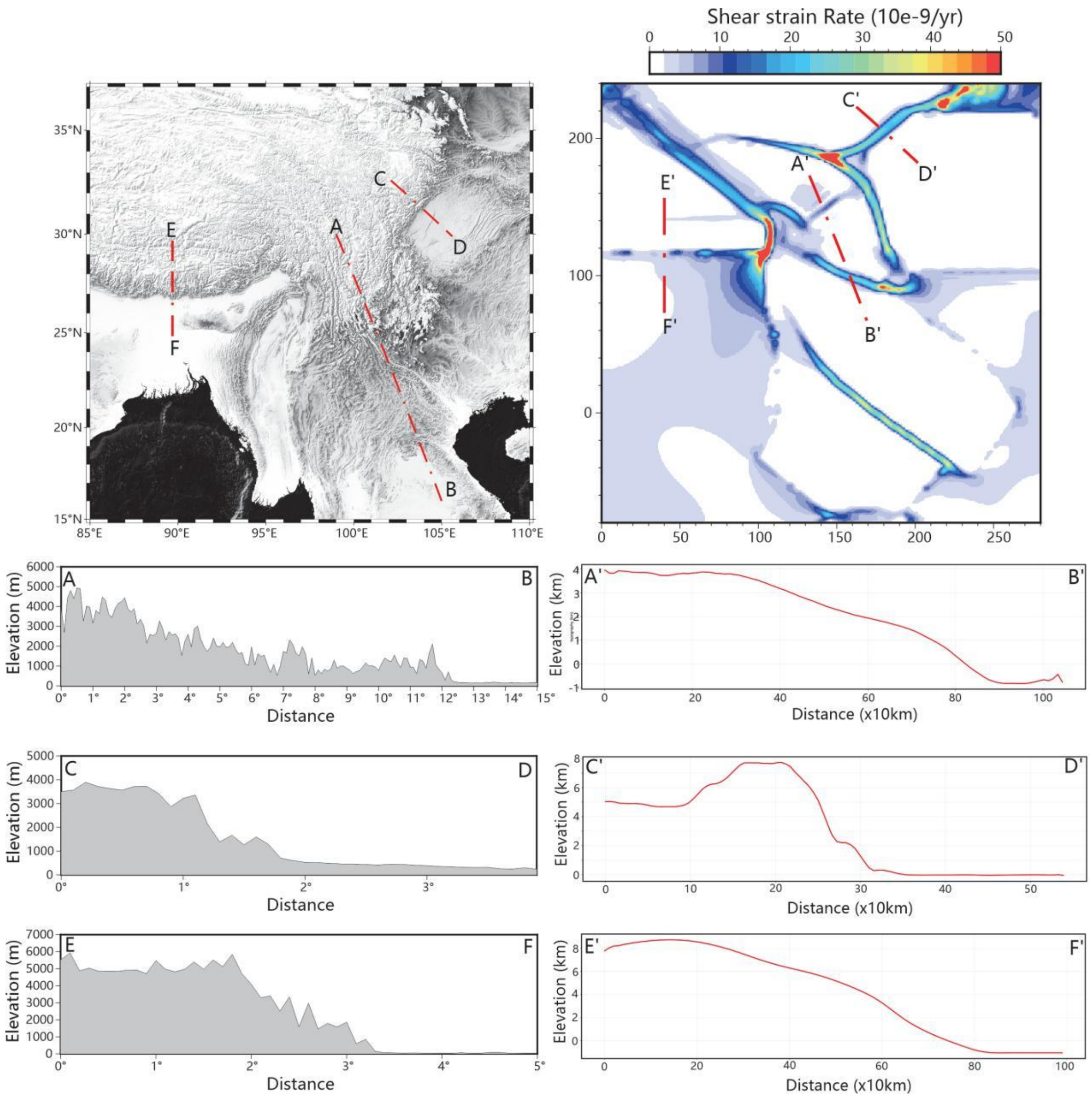

Figure S16 Comparison between model topography and actual topography.

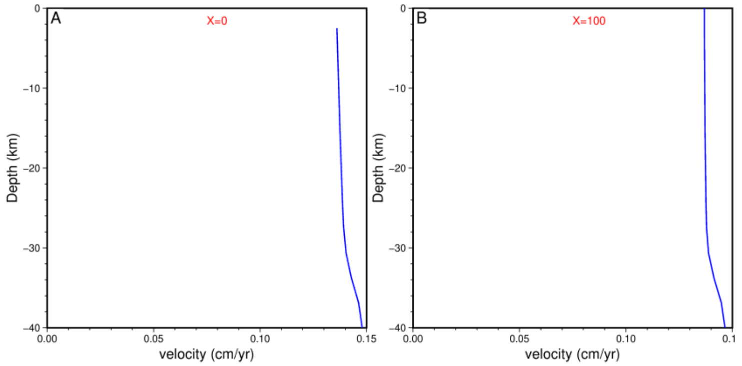

Figure S17 The velocity rates at different depths at 30Ma . Red letters indicate their selected positions on the X-axis of profile Fig. 5

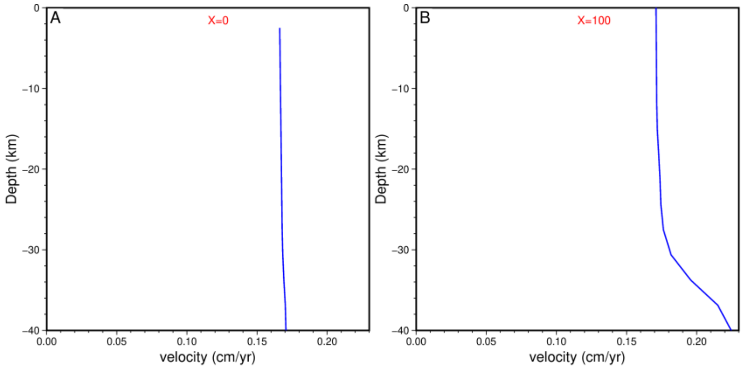

Figure S18 The velocity rates at different depths at 20Ma. Red letters indicate their selected positions on the X-axis of profile Fig. 5

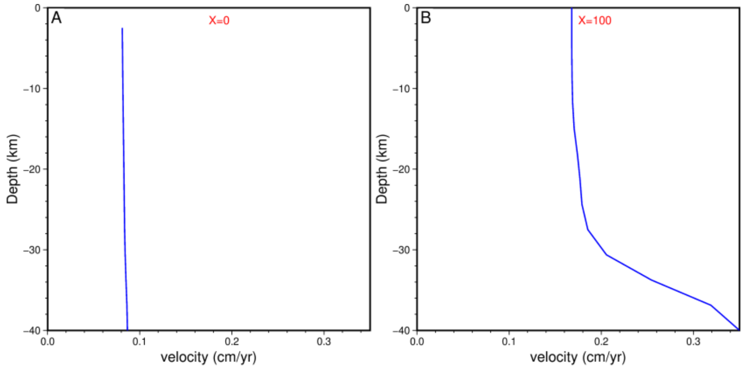

Figure S19 The velocity rates at different depths at 10Ma. Red letters indicate their selected positions on the X-axis of profile Fig. 5

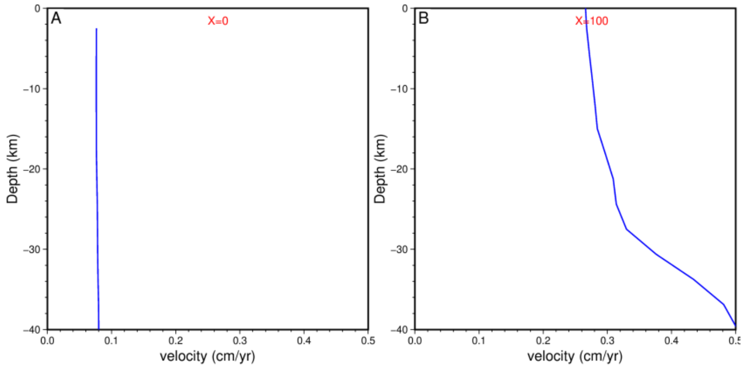

Figure S20 The velocity rates at different depths at present. Red letters indicate their selected positions on the X-axis of profile Fig. 5

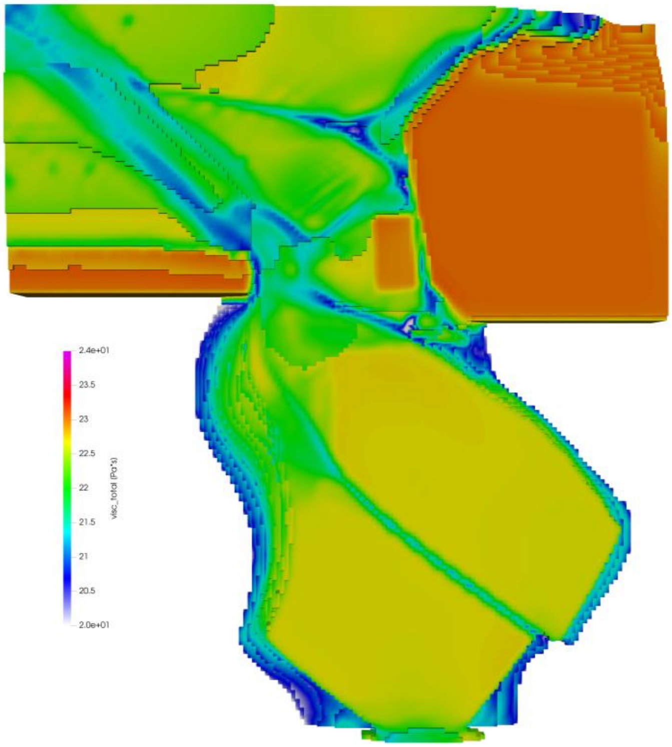

Figure S21 The viscosity distribution of the model simulating the Emeishan Large Igneous Province.

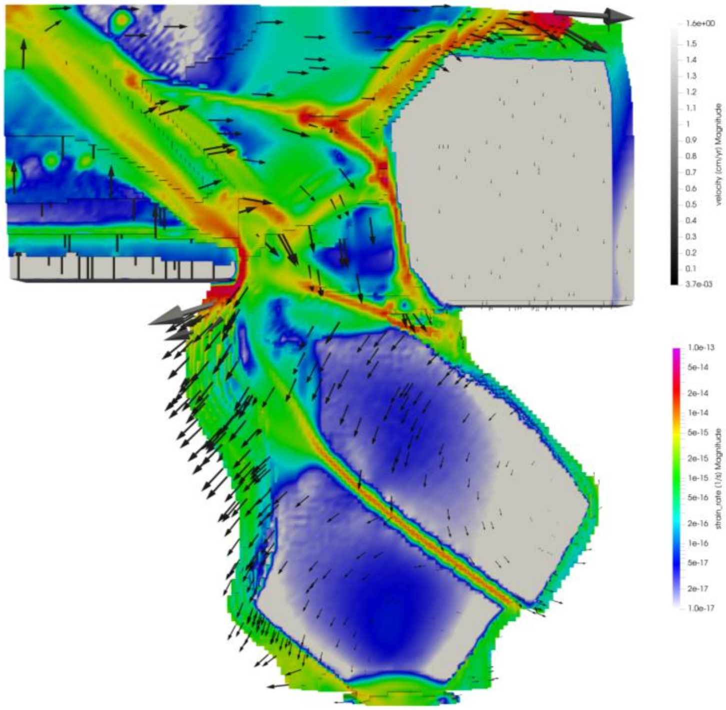

Figure S22 The strain rate and velocity distributions of the model simulating the Emeishan Large Igneous Province.

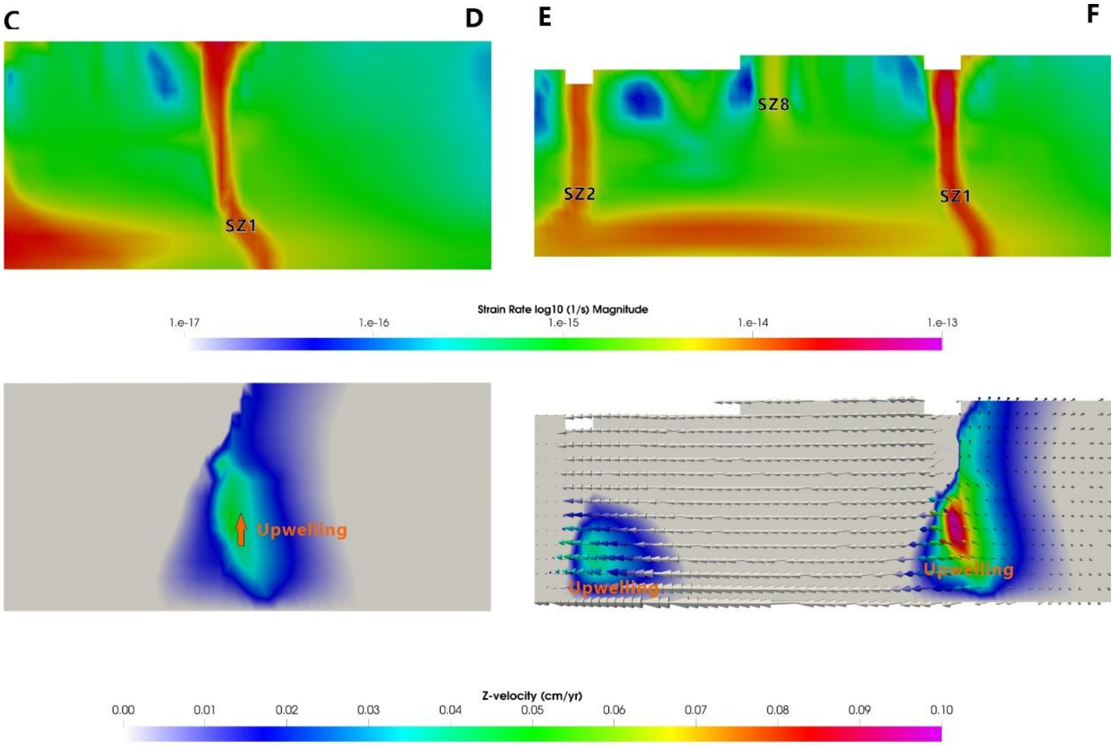

Figure S23 Upwelling of lower crustal materials around the shear zone periphery, Z-velocity represents the speed in the vertical direction, with positive values indicating an upward direction.

Table S1. Physical properties of rocks used in reference model

Material parameters	Upper crust[1]	Lower crust[2]	Strong block	Weak zone
Density, ρ_0($kg{\cdot}m^{-3}$)	2800	2900	2850	2800
Heat expansion, α (K^{-1})	3×10^{-5}	3×10^{-5}	3×10^{-5}	3×10^{-5}
Specific heat, Cp($kJ\ kg^{-1}\ K^{-1}$)	1.2	1.2	1.2	1.2
Heat conductivity, k ($W\ K^{-1}\ m^{-1}$)	2.5	2.5	2.5	2.5
Heat productivity, A ($\mu\ Wm^{-3}$)	1.0	0.3	0	0
Friction angle,Φ (°)	30→3	30→3	30	1
Cohesion, C_0 (MPa)	20	20	∞	1
Elastic shear modulus, G (GPa)	40	40	100	40
Creep pre-exponential factor, B_n ($Pa^{-n}\ s^{-1}$)	3.16×10^{-26}	3.2×10^{-20}	-	3.16×10^{-26}
Creep activation energy, E ($kJ\ mol^{-1}$)	190	276	-	190
Creep activation volume, V ($cm^3\ mol^{-1}$)	0	0	-	0
Power law exponent, n	3.3	3.0	-	3.3

The viscosity is computed by creep law : eII = Bn*Tau^n*exp(-(E + P*Vn)/(R*T)), eII=strain rate(1/s); Tau=stress(Pa); P=pressure(Pa); R=gas constant; Bn,n,E,V are given in table S1.Initial temperature-dependent density: $\rho_{P,T} = \rho_0[1-\alpha\ (T-T0)]$, where ρ0 is the reference density at temperature T_0. We adopt a linear plastic weakening of the friction angle, which decreases between plastic strains of 0.5 and 0.9. [1,2]Flow laws parameters are from Schmalholz (2009). The strong blocks (the indenter and the stable block) both use a high viscosity of 10^{24} Pa·s, while the free space uses a low viscosity of 10^{19} Pa·s.

Table S2. boundary conditions of tested models

Model[1]	Upper crust Thickness (km)	Lower crust thickness (km)	Free space viscosity (Pa·s)	Boundary temperature (℃)	convergence rate (cm/yr)
T700-U20-L20-F19-3	20	20	1e19	700	3
T700-U30-L10-F19-3	30	10	1e19	700	3
T700-U10-L30-F19-3	10	30	1e19	700	3
T800-U30-L10-F19-3	30	10	1e19	800	3
T800-U10-L30-F19-3	10	30	1e19	800	3
T800-U20-L20-F19-3	20	20	1e19	800	3
T600-U30-L10-F19-3	30	10	1e19	600	3
T600-U10-L30-F19-3	10	30	1e19	600	3
T600-U20-L20-F19-3	20	20	1e19	600	3
T700-U20-L20-F18-3	20	20	1e18	700	3
T700-U30-L10-F18-3	30	10	1e18	700	3
T700-U10-L30-F18-3	10	30	1e18	700	3
T700-U20-L20-F20-3	20	20	1e20	700	3
T700-U30-L10-F20-3	30	10	1e20	700	3
T700-U10-L30-F20-3	10	30	1e20	700	3
T700-U20-L20-F19-4	20	20	1e19	700	4
T700-U20-L20-F19-2	20	20	1e19	700	2

1.The model's name includes boundary conditions, where T = boundary temperature, U = upper crustal thickness, L = lower crustal thickness, F = free-space viscosity, and the last number means convergence rate. The blue color indicates the recommended model conditions.

Table S3. Timing and deformation of typical shortening structures

**Area/location**	**Structure**	**Timing of Cenozoic shortening**	**Dating method**	**References**
Chuxiong basin	NW-SE/NNW-SSE trend anticline-syncline pairs	~35Ma	Magnetic stratigraphy and 40Ar/39Ar dating on synsedimentary structures	Burchfiel & Chen, 2013; S. Li et al., 2020
Lanping-Simao fold belt	NW-SE/NNW-SSE trend anticline-syncline pairs	Eocene-early Oligocene(~30Ma)	Synsedimentary structures; AHe analysis	Burchfiel & Chen, 2013; Wang et al., 2020a
Jianchuan basin/Sichuan-Yunnan block (SW China)	long-wavelength, broad folds; NW-SE trend Ludian-Zhonghejiang thrust belt	50-39Ma	AHe and AFT analysis	Cao et al., 2021
Jianchuan basin/Sichuan-Yunnan block (SW China)	long-wavelength, broad folds; NE-SW trend Yulong thrust belt	28-20Ma	AHe and AFT analysis	Cao et al., 2019
Ninglang basin	broad syncline-anticline pair N-S trend Chenghai fold and thrust belt	Late Eocene -Oligocene	AHe and AFT analysis	Ma et al., 2021; Wang et al., 2025
Ailaoshan-Red River shear zone	NE- or NNE-striking	< 40Ma	Metamorphic zircon U-Pb data;	S. Cao et al., 2011; P. Searle et al.,

	folds of gneissic and migmatitic foliations		Granite U-Pb data	2010; H. Wang et al., 2019
Stong metamorphic complexes/Malaysia	NE- striking folds of schist and greenschist- to amphibolite-facies foliations	Eocene to Oligocene	ZHe, AHe and AFT analysis	Cottam et al., 2013; François et al., 2017
Khanom metamorphic complex	N-S oriented stretching lineation and migmatitic gneiss foliations	Paleogene	Zircon U-Pb data and monazite chemical Th–U-total Pb isochron method data	Kawakami et al., 2014; Sautter et al., 2019
Phuquoc-Kampot Som Basin/Gulf of Thailand	NW-SE folds and thrust faults	Paleocene to early Eocene	Synsedimentary structures; Zircon U-Pb data; AFT and ZFT analysis	Fyhn et al., 2010, 2016
Doi Inthanon metamorphic complex	N-S oriented boudin and folds; gneissic and migmatitic foliations are folded; asymmetric shear sense	~40Ma	Zircon and monazite U-Pb data	Barr et al., 2002; Macdonald et al., 2010; Morley, 2012; Wang et al., 2022b
Mogok metamorphic complex	N-S oriented amphibolite-granulite foliations are folded	43-32 Ma	monazite U-Pb ages	M.P. Searle et al., 2007, 2017; Lamont et al., 2021

Gaoligong shear zone	Tight to isoclinal folds; N-S, and NWN-SES oriented trending foliations	Early Cenozoic to the Late Oligocene	zircon U-Pb ages;	Song et al., 2010; B. Zhang et al., 2012
Khorat basin	NNE-SSW and NW-SE trend strike-slip faults inversion anticlines	50-45 Ma	synsedimentary structures; AFT analysis	Upton, 2000; Booth & Sattayarak, 2011; Morley, 2012
Mae Ping and Three Pagodas Fault zones	folds and uplift Eocene-Early Oligocene mylonitic mid-lower crust	45-30Ma	Zircon and monazite U-Pb data	Lacassin et al., 1998; Morley et al., 2007; Österle et al., 2019

Movie S1 (separate file). Strain rate and velocity evolution of recommended models.

SI References

1. Bai, D. et al. Crustal deformation of the eastern Tibetan plateau revealed by magnetotelluric imaging. Nat. Geosci. 3, 358–362 (2010).

2. Bao, X. et al. Two crustal low-velocity channels beneath SE Tibet revealed by joint inversion of Rayleigh wave dispersion and receiver functions. Earth and Planetary Science Letters 415, 16–24 (2015).

3. Barr, S. et al. New U-Pb and 40Ar/39Ar ages from the Doi Inthanon and Doi Suthep metamorphic core complexes, northwestern Thailand. Proc. Symp. Geol. Thail. 284–294 (2002).

4. Booth, J. & Sattayarak, N. Subsurface Carboniferous-Cretaceous geology of NE Thailand. Geol. Thail. 185–222 (2011).

5. Burchfiel, B. C. & Chen, Z. Tectonics of the Southeastern Tibetan Plateau and Its Adjacent Foreland. vol. 210 164 (2013).

6. Cao, K. et al. Thrusting, exhumation, and basin fill on the western margin of the South China block during the India-Asia collision. GSA Bulletin 133, 74–90 (2021).

7. Cao, K. et al. Oligocene-Early Miocene Topographic Relief Generation of Southeastern Tibet Triggered by Thrusting. Tectonics 38, 374–391 (2019).

8. Cao, S. et al. Oligo-Miocene shearing along the Ailao Shan-Red River shear zone: Constraints from structural analysis and zircon U/Pb geochronology of magmatic rocks in the Diancang Shan massif, SE Tibet, China. Gondwana Res. 19, 975–993 (2011).

9. Clark, M. K. & Royden, L. H. Topographic ooze: Building the eastern margin of Tibet by lower crustal flow. Geology 28, 703 (2000).

10. Cottam, M. A., Hall, R. & Ghani, A. A. Late cretaceous and cenozoic tectonics of the Malay Peninsula constrained by thermochronology. J. Asian Earth Sci. 76, 241–257 (2013).

11. François, T. et al. Late Cretaceous extension and exhumation of the Stong and Taku magmatic and metamorphic complexes, NE Peninsular Malaysia. J. Asian Earth Sci. 143, 296–314 (2017).

12. Fyhn, M. B. W. et al. Cenozoic deformation and exhumation of the Kampot Fold Belt and implications for south Indochina tectonics. Journal of Geophysical Research: Solid Earth 121, 5278–5307 (2016).

13. Fyhn, M. B. W. et al. Palaeocene-early Eocene inversion of the Phuquoc-Kampot Som Basin: SE Asian deformation associated with the suturing of Luconia. J. Geol. Soc. 167, 281–295 (2010).

14. Gan, W. et al. Initiation of Clockwise Rotation and Eastward Transport of Southeastern Tibet Inferred from Deflected Fault Traces and GPS Observations. GSA Bull. 134, 1129–1142 (2022).

15. Gan, W. et al. Present-day crustal motion within the Tibetan Plateau inferred from GPS measurements. J. Geophys. Res.: Solid Earth 112, 2005JB004120 (2007).

16. HaoBo, W. et al. Cenozoic multi-metamorphism, shear deformation and geological significance of Ailaoshan high-grade metamorphic complex, western Yunnan, China. Acta Petrol. Sin. 35, 2573–2596 (2019).

17. Kawakami, T. et al. U-Pb zircon and CHIME monazite dating of granitoids and high-grade metamorphic rocks from the Eastern and Peninsular Thailand - A new report of Early Paleozoic granite. Lithos 200–201, 64–79 (2014).

18. Kreemer, C. Absolute plate motions constrained by shear wave splitting orientations with implications for hot spot motions and mantle flow. J. Geophys. Res.: Solid Earth 114, 2009JB006416 (2009).

19. Kreemer, C., Hammond, W. C. & Blewitt, G. A Robust Estimation of the 3-D Intraplate Deformation of the North American Plate From GPS. J. Geophys. Res.: Solid Earth 123, 4388–4412 (2018).

20. Lacassin, R., Replumaz, A. & Hervé Leloup, P. Hairpin river loops and slip-sense inversion' on southeast Asian strike-slip faults. Geology 26, 703 (1998).

21. Lamont, T. N. et al. Late Eocene-Oligocene granulite facies garnet-sillimanite migmatites from the Mogok Metamorphic belt, Myanmar, and implications for timing of slip along the Sagaing Fault. Lithos 386–387, 106027 (2021).

22. Li, S. et al. Oligocene Deformation of the Chuandian Terrane in the SE Margin of the Tibetan Plateau Related to the Extrusion of Indochina. Tectonics 39, e2019TC005974 (2020).

23. Li, Y., Shan, X., Gao, Z. & Huang, X. Interseismic Coupling, Asperity Distribution, and Earthquake Potential on Major Faults in Southeastern Tibet. Geophys. Res. Lett. 50, e2022GL101209 (2023).

24. Li, Z. & Kreemer, C. Eastward mantle flow field underneath East Asia quantified by combining shear-wave splitting orientations and absolute plate motion observations. Earth Planet. Sci. Lett. 566, 116969 (2021).

25. Li, Z. et al. Diffuse Deformation in the SE Tibetan Plateau: New Insights From Geodetic Observations. JGR Solid Earth 125, e2020JB019383 (2020).

26. Ma C., Ye Z., Cao Y. & Wang C. Formation and evolution of Cenozoic basin in Ninglang of Yunnan Province. Geological Survey of China 8, 80–88 (2021).

27. Macdonald, A. S. et al. P-T-t constraints on the development of the Doi Inthanon metamorphic core complex domain and implications for the evolution of the western gneiss belt, northern Thailand. Journal of Asian Earth Sciences 37, 82–104 (2010).

28. Morley, C. K. Variations in Late Cenozoic-Recent strike-slip and oblique-extensional geometries, within Indochina: The influence of pre-existing fabrics. J. Struct. Geol. 29, 36–58 (2007).

29. Morley, C. K. Late Cretaceous-Early Palaeogene tectonic development of SE Asia. Earth-Science Reviews 115, 37–75 (2012).

30. Österle, J. E., Klötzli, U., Stockli, D. F., Palzer-Khomenko, M. & Kanjanapayont, P. New age constraints on the Lan Sang gneiss complex, Thailand, and the timing of activity of the Mae Ping shear zone from in-situ and depth-profile zircon and monazite U-Th-Pb geochronology. J. Asian Earth Sci. 181, 103886 (2019).

31. Sautter, B. et al. Exhumation of west Sundaland: A record of the path of India? Earth Sci. Rev. 198, 102933 (2019).

32. Savage, J. C. & Burford, R. O. Geodetic determination of relative plate motion in central California. J. Geophys. Res. 78, 832–845 (1973).

33. Schmalholz, S. M., Kaus, B. J. P. & Burg, J.-P. Stress-strength relationship in the lithosphere during continental collision. Geology 37, 775–778 (2009).

34. Searle, M. P. et al. Tectonic evolution of the Mogok metamorphic belt, Burma (Myanmar) constrained by U-Th-Pb dating of metamorphic and magmatic rocks. Tectonics 26, 2006TC002083 (2007).

35. Searle, M. P., Yeh, M.-W., Lin, T.-H. & Chung, S.-L. Structural constraints on the timing of left-lateral shear along the Red River shear zone in the Ailao Shan and Diancang Shan Ranges, Yunnan, SW China. Geosphere 6, 316–338 (2010).

36. Searle, M. P. et al. Tectonic and Metamorphic Evolution of the Mogok Metamorphic and Jade Mines Belts and Ophiolitic Terranes of Burma (Myanmar). vol. 48 293 (2017).

37. Song, S., Niu, Y., Wei, C., Ji, J. & Su, L. Metamorphism, anatexis, zircon ages and tectonic evolution of the Gongshan block in the northern Indochina continent-An eastern extension of the Lhasa Block. Lithos 120, 327–346 (2010).

38. Upton, D. R. A regional fission track study of Thailand: implications for thermal history and denudation. (Birkbeck (University of London), 2000).

39. Wang, M. & Shen, Z.-K. Present-Day Crustal Deformation of Continental China Derived From GPS and Its Tectonic Implications. Journal of Geophysical Research: Solid Earth 125, (2020).

40. Wang, Y. et al. Cenozoic Exhumation of the Ailaoshan-Red River Shear Zone: New Insights From Low-Temperature Thermochronology. Tectonics 39, e2020TC006151 (2020).

41. Wang, Y. et al. Kinematics and 40Ar/39Ar geochronology of the Lincang-Inthanon tectonic belt: Implication for Cenozoic tectonic extrusion of SE Asia. GSA Bull. 134, 2854–2866 (2022).

42. Wang, Y. et al. Cenozoic Crustal Shortening and Structural Transition in the NW Yunnan Basin Region, SE Tibetan Plateau. Tectonics 44, e2024TC008688 (2025).

43. Yang, J. et al. Lower Crustal Rheology Controls the Development of Large Offset Strike-Slip Faults During the Himalayan-Tibetan Orogeny. Geophys. Res. Lett. 47, e2020GL089435 (2020).

44. Zhang, B. et al. Polystage deformation of the Gaoligong metamorphic zone: Structures, 40Ar/ 39Ar mica ages, and tectonic implications. Journal of Structural Geology 37, 1–18 (2012).
